# A novel prospective isolation of murine fetal liver progenitors to study *in utero* hematopoietic defects

**DOI:** 10.1371/journal.pgen.1007127

**Published:** 2018-01-04

**Authors:** Julia E. Draper, Patrycja Sroczynska, Muhammad Z. H. Fadlullah, Rahima Patel, Gillian Newton, Wolfgang Breitwieser, Valerie Kouskoff, Georges Lacaud

**Affiliations:** 1 Cancer Research UK Stem Cell Biology Group, Cancer Research UK Manchester Institute, Manchester Cancer Research Centre, The University of Manchester, Manchester, United Kingdom; 2 Biotech Research and Innovation Centre, University of Copenhagen, Copenhagen, Denmark; 3 Centre for Epigenetics, University of Copenhagen, Copenhagen, Denmark; 4 Molecular Biology Core Facility, Cancer Research UK Manchester Institute, Manchester Cancer Research Centre, The University of Manchester, Manchester, United Kingdom; 5 Division of Developmental Biology & Medicine, Michael Smith Building, The University of Manchester, Manchester, United Kingdom; Cincinnati Children's Hospital Medical Center, UNITED STATES

## Abstract

In recent years, highly detailed characterization of adult bone marrow (BM) myeloid progenitors has been achieved and, as a result, the impact of somatic defects on different hematopoietic lineage fate decisions can be precisely determined. Fetal liver (FL) hematopoietic progenitor cells (HPCs) are poorly characterized in comparison, potentially hindering the study of the impact of genetic alterations on midgestation hematopoiesis. Numerous disorders, for example infant acute leukemias, have *in utero* origins and their study would therefore benefit from the ability to isolate highly purified progenitor subsets. We previously demonstrated that a *Runx1 distal promoter (P1)-GFP*::*proximal promoter (P2)-hCD4* dual-reporter mouse (*Mus musculus*) model can be used to identify adult BM progenitor subsets with distinct lineage preferences. In this study, we undertook the characterization of the expression of *Runx1-P1-GFP* and *P2-hCD4* in FL. Expression of *P2-hCD4* in the FL immunophenotypic Megakaryocyte-Erythroid Progenitor (MEP) and Common Myeloid Progenitor (CMP) compartments corresponded to increased granulocytic/monocytic/megakaryocytic and decreased erythroid specification. Moreover, *Runx1-P2-hCD4* expression correlated with several endogenous cell surface markers’ expression, including CD31 and CD45, providing a new strategy for prospective identification of highly purified fetal myeloid progenitors in transgenic mouse models. We utilized this methodology to compare the impact of the deletion of either total RUNX1 or RUNX1C alone and to determine the fetal HPCs lineages most substantially affected. This new prospective identification of FL progenitors therefore raises the prospect of identifying the underlying gene networks responsible with greater precision than previously possible.

## Author summary

The production of red blood cells, platelet-producing megakaryocytes, and immune response-directing granulocytes and monocytes is initiated at an early stage in the developing embryo and continues throughout life. The proportion of each cell type varies depending on the specific needs of the organism. We know that in the mouse embryo, specialized blood progenitor cells emerge in the fetal liver and produce mature blood cells in response to different cues. However, it is difficult to distinguish between red blood cell and white blood cell-producing progenitors with sufficiently high accuracy to study these cues. For example, we know that several childhood blood disorders, such as leukemias, are caused by genetic mutations in blood progenitor cells before birth, but studying the effects of these mutations in a mouse disease model is hampered if we don’t know which blood progenitor cells to collect. We have used different genetic markers to help distinguish red blood cell, megakaryocyte and granulocyte/monocyte-producing progenitor cells with a greater precision than was previously possible. Furthermore, to illustrate how this technique can be used to study blood disorders, we demonstrated that mutations affecting the transcription factor *Runx1* impair the abilities of different progenitors to produce mature blood cells in different ways.

## Introduction

Definitive hematopoiesis is a complex, multistep process involving increasingly restrictive cell fate decisions by self-renewing, multipotent hematopoietic stem cells (HSCs). Commitment to lymphoid, granulocytic/monocytic (GM), megakaryocytic and erythroid lineages occurs through the differentiation of immature progenitors, and is subject to spatial and temporal control by intrinsic and extrinsic factors [1,2]. In recent years, high-resolution characterization of BM progenitor populations has been achieved and this critical advance has allowed detailed interrogation of the gene regulatory networks which govern normal homeostatic and malignant hematopoietic differentiation, both in clinical patient samples and adult transgenic mouse models [3–16]. In particular, various assumptions about the process of myeloid progenitor differentiation have been challenged. For example, the existence of an obligatory intermediate CMP population, as an ancestor of all committed granulocytic/monocytic, megakaryocytic and erythroid progenitors, is now questioned [3,7]. Pronk *et al* proposed the separation of bone marrow CMPs into PreGM and PreMegakaryocytic/Erythroid (PreMegE) progenitors, based on CD150/Endoglin expression [5]. Utilizing a *Runx1* dual-reporter mouse model (*P1-GFP*::*P2-hCD4*) [17] (which reflects the alternate use of the two *Runx1* promoters) we recently reported that in fact the *P2-hCD4*^*-*^ PreMegE fraction comprises pro-erythroid progenitors, whereas *P2-hCD4*^*+*^ PreMegEs are skewed in favor of megakaryocytic output [18].

Examinations of the roles of developmental transcription factors (TFs), particularly *Runx* factors, in the developing mouse embryo have greatly advanced our understanding of the origins of blood development *in utero* [19,20]. *Runx1* expression is observed in the different hematopoietic waves: in the earliest primitive myeloid and erythroid progenitors at embryonic day (E) 7.5, in the intermediate erythromyeloid progenitors (EMPs)/lymphoid progenitors at E8.5, and in the long-term repopulating HSCs from E10.5 [21–27]. Indeed, HSC emergence through endothelial-to-hematopoietic transition (EHT) is directed by *P2-*expressed RUNX1B [17,28].

The fetal liver is seeded initially by EMPs, followed by definitive HSCs from E11.5, and forms an ideal niche for hematopoietic stem and progenitor cell (HSPC) expansion and maturation [21,29,30]. By E14.5, a definitive hematopoietic stem and progenitor cell hierarchy is believed to be firmly established in the fetal liver [31,32]. Equivalents of previously described BM HPCs, including MEPs, CMPs, Granulocyte/Monocyte Progenitors (GMPs) and Megakaryocyte Progenitors (MkPs), have been identified in E14.5 fetal liver [31,33]. However, the resolution at which these fetal liver hematopoietic progenitor cells can be identified and isolated is far lower than for their bone marrow equivalents [9,31,34–37]. This hinders the interrogation of this fetal liver hierarchy, particularly in mouse models of hematopoietic disorders with *in utero* origins. Indeed, higher-resolution purification of myeloid hematopoietic progenitor cells would allow the execution of transcriptomic and clonogenic lineage analysis to investigate the disruption of specific gene regulatory networks and the resultant impact on hematopoietic output. We therefore endeavored to improve our resolution of the specification of fetal liver myeloid hematopoietic progenitor cells.

Having initially determined that multilineage cultured colony-forming unit (CFU-C) activity is restricted to the CD55^+^ CD150^+^ Megakaryocyte-Erythroid Progenitor (MEP) and Common Myeloid Progenitor (CMP) compartments, we utilized the *Runx1-P1-GFP*::*P2-hCD4* mouse model to further subdivide these populations. Within the MEP, the *P2-hCD4*^*+*^ fraction possessed the entirety of its bipotential megakaryocytic/erythroid (Mk/Ery) output. The *P2-hCD4*^*+*^ CMP, meanwhile, had more balanced myeloid output, in particular decreased erythroid specification, than its *P2-hCD4*^*-*^ counterpart.

Subsequently, we identified CD31, CD45 and CD48 as candidate markers whose expression correlated with *P2-hCD4*. To demonstrate the potential applications of this approach, we characterized perturbations in CD150^+^ CD31^-/+^ MEPs and CD150^+^ CD31^low/high^ CMPs from *Runx1*-null (*Runx1-flox::Vav1-Cre, Runx1-del[38]*) and *Runx1c*-null (*Runx1-P1-MRIPV* [39]) mice. Using this approach we revealed fundamental differences between the two mouse models. Whereas, *Runx1-del/del* CD31^low^ CMPs have impaired erythroid specification and maturation, *Runx1*-*P1-MRIPV/MRIPV* CD31^high^ CMPs have impaired megakaryocytic specification but normal megakaryocyte and erythroid maturation. This indicates that the RUNX1B and RUNX1C isoforms fulfill different roles during fetal liver hematopoiesis. Furthermore, it demonstrates the benefits of high specificity fetal liver hematopoietic progenitor isolation for elucidating complex myeloid lineage fate decisions.

## Results

### Delineating CD150/CD55 expression allows enhanced purification of immunophenotypic MEP and CMP fractions in E14.5 fetal liver

**Fig 1:**
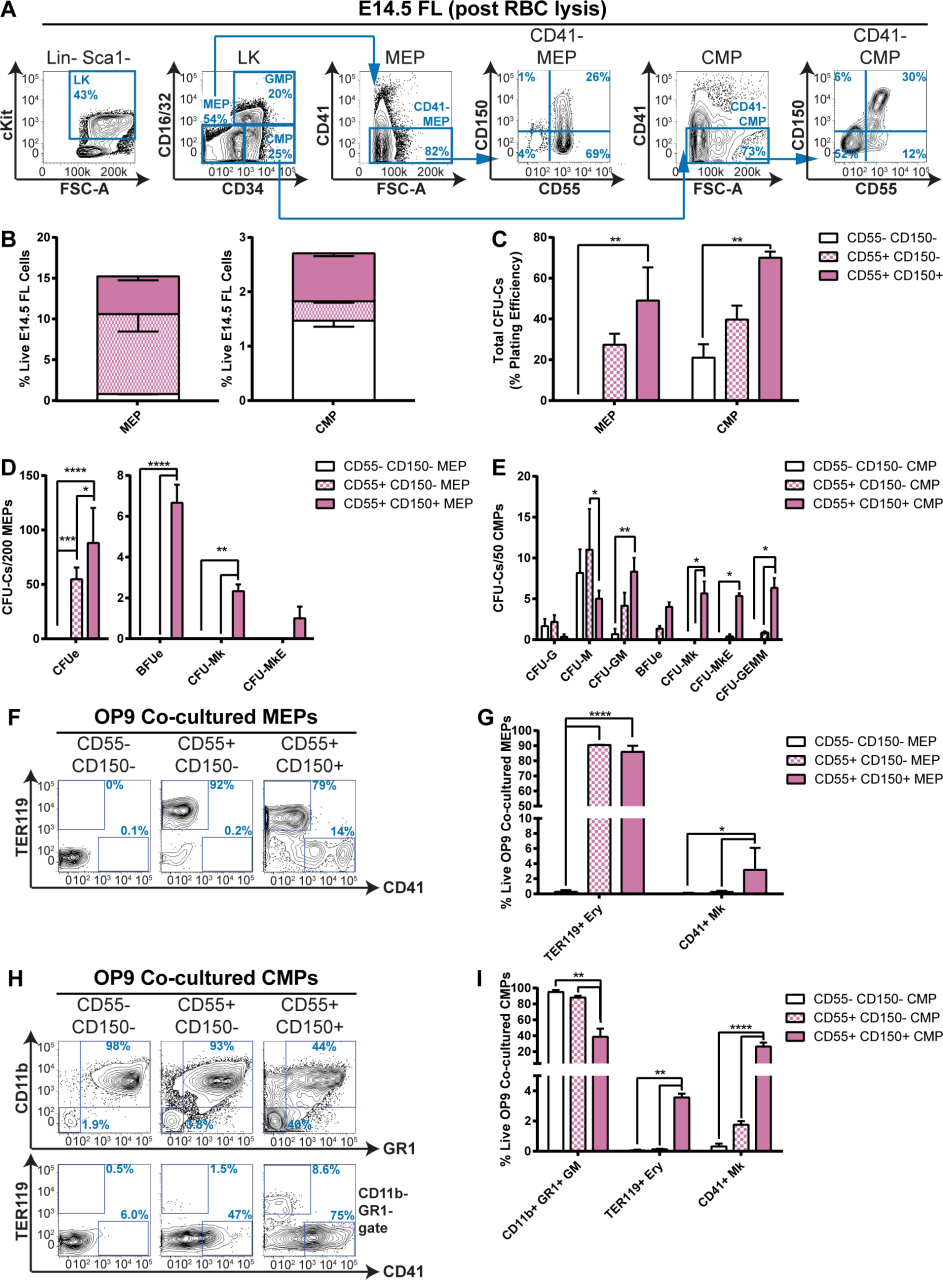
Delineating CD150/CD55 expression allows enhanced purification of immunophenotypic MEP and CMP fractions in E14.5 fetal liver. **A-B**. Expression of CD150 and CD55 in wild type (WT) E14.5 fetal liver immunophenotypic MEP and CMP fractions. A. Representative FACS plots of Lin^-^ Sca1^-^ cKit^+^ (LK) hematopoietic progenitors (1^st^ panel); LK hematopoietic progenitors: CD16/32^low^ CD34^-^ MEPs, CD16/32^low^ CD34^+^ CMPs and CD16/32^high^ CD34^+^ GMPs (2^nd^ panel); CD41^-^ MEPs (3^rd^ panel); CD150/CD55 expression in MEPs (4^th^ panel); CD41^-^ CMPs (5^th^ panel);; and CD150/CD55 expression in CMPs (6^th^ panel). B. Quantitation of the proportion of CD55^-^ CD150^-^, CD55^+^ CD150^-^ and CD55^+^ CD150^+^ MEPs (left) and CMPs (right) as a percentage of total live red blood cell lysed fetal liver cells. N = 3 **C-E**. CFU-C activity of CD55^-^ CD150^-^, CD55^+^ CD150^-^ and CD55^+^ CD150^+^ MEPs and CMPs. C. Total CFU-C numbers per 100 plated cells. D. Differential (CFUe, BFUe, CFU-Mk and CFU-MkE) activity of MEPs. E. Differential (CFU-G, CFU-M, CFU-GM, BFUe, CFU-Mk, CFU-MkE and CFU-GEMM) activity of CMPs. N = 3 **F-I**. Lineage output of OP9 co-cultured MEPs and CMPs. F. Representative FACS plots of TER119 and CD41 expression of day 7 OP9 co-cultured MEPs. G. Proportion of TER119^+^ erythroid cells and CD41^+^ megakaryocyte cells in day 7 OP9 MEP co-cultures. H. Representative FACS plots of CD11b, GR1, TER119 and CD41 expression of day 7 OP9 co-cultured CMPs. I. Proportion of CD11b^+^ GR1^+^ granulocyte/monocyte cells, TER119^+^ erythroid cells and CD41^+^ megakaryocyte cells in day 7 OP9 CMPs co-cultures. N = 3.

The existence of a bipotent MEP has long been posited in mid-gestation fetal liver, in addition to adult bone marrow [3,31]. The multipotent CMP is more controversial, however, with various groups reporting that it is actually a heterogeneous population of megakaryocytic/erythroid/granulocytic/monocytic progenitors, capable of varying lineage output [5,40]. More recently, CD150 and CD55 expression have been associated with Mk/Ery potential in adult hematopoietic progenitors [41]. Therefore, we analyzed CD55/CD150 expression in the E14.5 fetal liver MEP (Lin^-^ SCA1^-^ C-KIT^+^ (LK) CD16/32^low^ CD34^-^) and CMP (LK CD16/32^low^ CD34^+^) progenitor fractions (Fig 1A and 1B). Additionally, to remove the previously identified CD41^+^ MkP fraction [33,42], we analyzed solely CD41^-^ MEPs and CMPs.

In both the CD41^-^ MEP and CMP fractions, three populations were discernible: CD55^-^ CD150^-^ (4% of MEPs, 52% CMPs); CD55^+^ CD150^-^ (69% MEPs, 12% CMPs); and CD55^+^ CD150^+^ (26% MEPs, 30% CMPs). By contrast, we observed the GMP and MkP populations were more homogeneous with respect to both CFU-C activity (S1A Fig) and CD150/CD55 expression: 95% of MkPs were CD55^+^ CD150^+^ and 91% of GMPs were CD55^low^ CD150^low^ (S1B–S1F Fig). We also confirmed that negligible (<1%) CFU-C activity resided in the remaining cKit^-^ fetal liver fraction (S1A Fig). We therefore proceeded to characterize the differentiation potentials of the various CD55/CD150 MEP and CMP subfractions.

Interestingly, CD55^-^ CD150^-^ MEPs did not possess any CFU-C activity in semi-solid myeloid MethoCult medium (Fig 1C). Additionally, they did not yield cells expressing either the erythroid marker TER119, the megakaryocytic marker CD41, the granulocytic/monocytic markers CD11b/GR1 or the mast/progenitor cell-associated C-KIT following OP9 co-culture in pro-myeloid medium (Fig 1F and 1G, S1G and S1H Fig). By contrast, CD55^-^ CD150^-^ CMPs yielded CFU-Cs at 20% plating efficiency, which solely comprised granulocytic (CFU-G), monocytic/macrophage (CFU-M) and GM (CFU-GM) colonies (Fig 1C and 1E). Additionally, 98% of OP9 co-cultured CD55^-^ CD150^-^ CMP-derived cells were CD11b^+^ GR1^+^ granulocytes/monocytes (Fig 1H and 1I), with just 0.01% being TER119^+^ erythrocytes and 0.11% CD41^+^ megakaryocytes.

CD55^+^ CD150^-^ cells had increased CFU-C activity by comparison (25% for MEPs, 40% for CMPs, Fig 1C). CD55^+^ CD150^-^ MEPs solely comprised erythroid colony-forming units (CFUe, Fig 1D) and produced 92% TER119^+^ erythroid cells on OP9 (Fig 1F and 1G). CD55^+^ CD150^-^ CMPs chiefly yielded CFU-G, M and GM colonies, but also comprised a small number (approximately 1%) of erythroid burst-forming units (BFUe), bipotent megakaryocytic/erythroid colony-forming units (CFU-MkE) and multipotential granulocytic/erythroid/monocytic/megakaryocytic colony-forming units (CFU-GEMM, Fig 1E). Accordingly 93% CD11b^+^ GR1^+^ granulocytic/monocytic and 1.8% CD41^+^ megakaryocytic cells resulted from OP9 co-culture (Fig 1H ang 1I).

Finally, CFU-C activity was chiefly observed in the CD55^+^ CD150^+^ fractions (50% for MEPs, 70% for CMPs, Fig 1C). Crucially, the CD55^+^ CD150^+^ subpopulation was the only MEP fraction to demonstrate megakaryocytic/erythroid bipotentiality (yielding 1.2% CFU-Mks and 0.5% CFU-MkEs, Fig 1D; 79% TER119^+^ erythrocytes and 14% CD41^+^ megakaryocytes, Fig 1F and 1G). Additionally, the CD55^+^ CD150^+^ CMP fraction had the broadest range of CFU-C activity, yielding approximately equal numbers of CFU-M, GM, Mk, MkE, GEMM and BFUe colonies (Fig 1E) and all three Mk/Ery/GM cell fractions in OP9 co-culture (Fig 1H and 1I). We therefore demonstrated that inclusion of anti-CD150 and, to a lesser extent, anti-CD55 antibodies in MEP/CMP isolation protocols aids the purification of highly clonogenic multipotent myeloid progenitors.

The FL Lin^-^ cKit^high^ Sca1^high^ (LSK) is a highly clonogenic fraction, which can be separated into long-term repopulating HSC and short-term repopulating Multipotent Progenitor (MPP) fractions on the basis of CD48 and CD150 expression [32,43] (S1I and S1J Fig). To determine whether fetal liver LSK hematopoietic stem and progenitor cells are the likely ancestors of the fetal liver CMPs, MEPs, GMPs and MkPs, we cultured E14.5 fetal liver HSCs and LSK CD48^-/+^ MPPs in pro-myeloid medium for 20 hours (S1K and S1L Fig). The majority of cultured HSCs (60%) retained an LSK immunophenotype, but had upregulated CD48. Only 6% of cultured HSCs had an LK immunophenotype, but within this fraction CD150^+/-^ CMP, CD150^+/-^ MEP, GMP and MkP fractions were discernible. More convincingly, a large fraction (27%) of cultured LSK CD48^+^ CD150^+^ MPPs had acquired an LK immunophenotype, yielding mostly CD150^+^ MEPs and MkPs. By contrast, 12% of cultured LSK CD48^+^ CD150^-^ MPPs had an MkP, GMP, CD150^-^ MEP or CD150^-^ CMP immunophenotype. This therefore suggests that fetal liver HSCs are capable of establishing a myeloid progenitor hierarchy, as their LSK CD48^+^ MPP progeny give rise to the Sca1^-^ cKit^+^ MkP, GMP, MEP and CMP progenitors.

The observation that fetal liver HSCs are capable of establishing a hematopoietic stem and progenitor cell hierarchy does not preclude the possibility that yolk sac erythro-myeloid progenitors (EMPs) could also contribute to these cell populations. To determine whether EMPs could directly give rise to a hematopoietic progenitor cell profile comparable to that observed in the fetal liver, we isolated yolk sac cells from E9.5 embryos and cultured them in pro-myeloid medium for up to 24 hours (with or without prior explant culture (S1M and S1N Fig)). We observed that a small proportion of cells (up to 15% in the explant cultures) had the LK immunophenotype. The majority of these cells were either immunophenotypic GMPs or were LK CD16/32^-^ CD150^-^ CD41^+^. No CD150^+^ LK progenitors were produced in these cultures. This therefore suggests that yolk sac EMPs are incapable of directly establishing our observed FL myeloid hematopoietic progenitor hierarchy.

### *Runx1-P2* reporter expression demarcates mono and bipotent subsets in the CD150^+^ MEP

**Fig 2:**
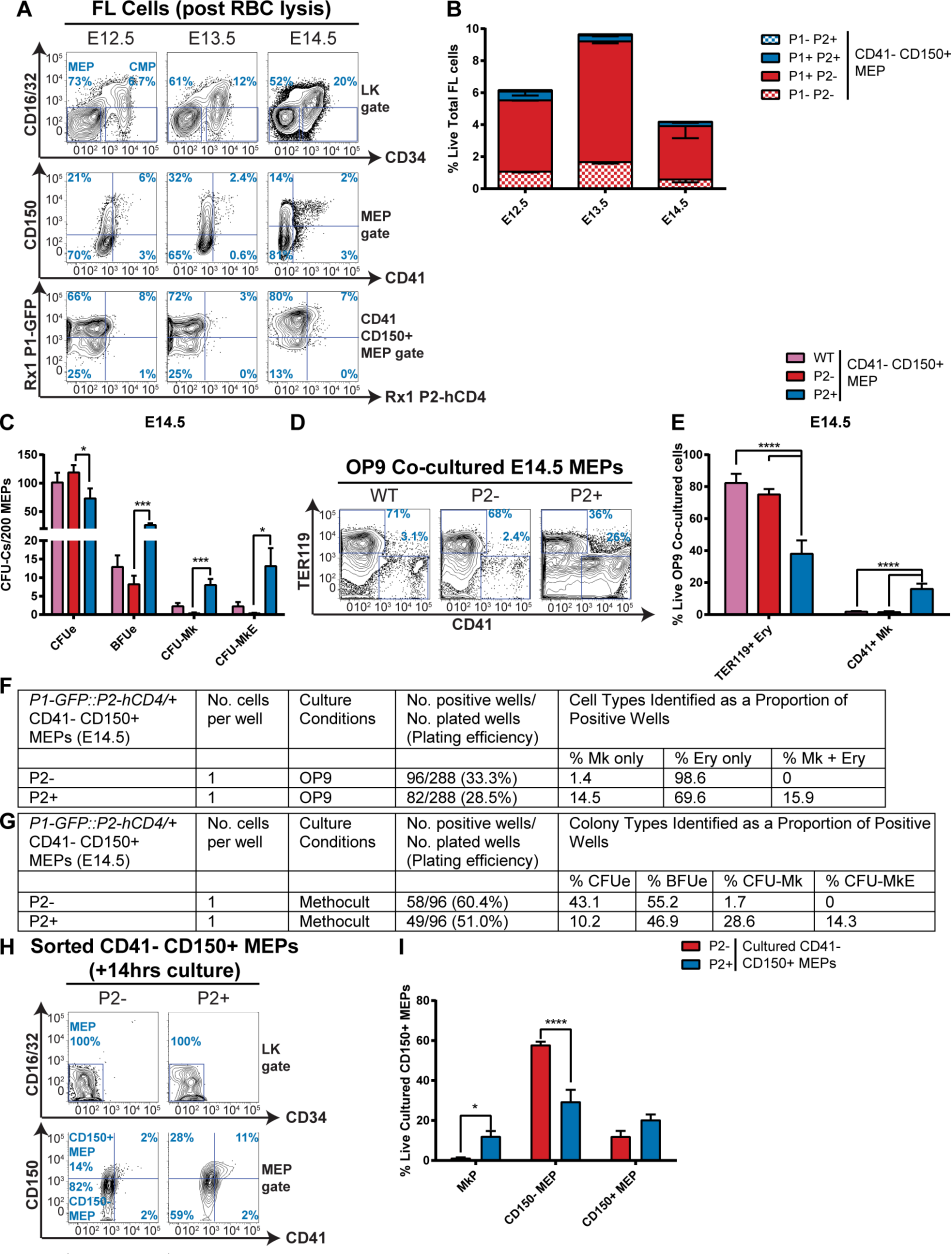
*Runx1-P2* reporter expression demarcates mono and bipotent subsets in the CD150^+^ MEP. **A-B.** Expression of *Runx1-P1-GFP* and *P2-hCD4* in *P1-GFP*::*P2-hCD4/+* E12.5, E13.5 and E14.5 FL CD41^-^ CD150^+^ MEPs. A. Representative FACS plots. B. Quantitation of the proportions of *P1*^*-*^ *P2*^*-*^, *P1*^*+*^ *P2*^*-*^, *P1*^*+*^ *P2*^*+*^ and *P1*^*-*^ *P2*^*+*^ MEPs as a percentage of total live red blood cell lysed fetal liver cells. N = 3 C. Differential CFU-C activity of wild type, *P2-hCD4*^*-*^ and *P2-hCD4*^*+*^ E14.5 FL CD41^-^ CD150^+^ MEPs. N = 6 **D-E.** Lineage output of day 7 OP9 co-cultured wild type, *P2-hCD4*^*-*^ and *P2-hCD4*^*+*^ CD41^-^ CD150^+^ MEPs. D. Representative FACS plots of TER119 and CD41. E. Proportion of erythroid and megakaryocyte cells. N = 4 **F-G.** Lineage output of single cultured *P2*^*-*^ and *P2*^*+*^ MEPs in OP9 co-cultures (F) and MethoCult semi-solid myeloid culture medium (G). **H-I.** Short-term (14 hours) differentiation of *P2*^*-*^ and *P2*^*+*^ CD41^-^ CD150^+^ MEPs in pro-myeloid liquid culture. H. Representative FACS plots of CD16/32/CD34 (top) and CD150/CD41 (bottom) expression in the LK gate of cultured MEPs. I. Proportions of immunophenotypic MkP, CD41^-^ CD150^-^ MEPs and CD41^-^ CD150^+^ MEPs in short-term cultures. N = 5.

We previously used *Runx1-P1-GFP*::*P2-hCD4* dual-reporter mice to demonstrate that *Runx1-P2* expression coincides with enhanced megakaryocytic specification in adult bone marrow PreMegEs [18]. Having established that CD150 expression enhances megakaryocyte/erythroid progenitor isolation in fetal liver MEPs, we tried to improve this further by characterizing *Runx1-P1-GFP/P2-hCD4*-expressing MEPs (Fig 2A and 2B). In E12.5, E13.5 and E14.5 fetal liver, the CD41^-^ CD150^+^ MEP fractions were dominated by *P1-GFP*^*+*^ *P2-hCD4*^*-*^ cells (66%, 72% and 80% respectively). *P1-GFP*^*-*^ *P2-hCD4*^*-*^ populations were also present (25%, 25% and 13% respectively). However, *P1-GFP*^*+*^ *P2-hCD4*^*+*^ populations were considerably smaller, at 8%, 3% and 7% of CD41^-^ CD150^+^ MEPs respectively. Importantly, at each timepoint this restricted *P2-hCD4*^*+*^ fraction possessed the vast majority of BFUe, CFU-Mk and CFU-MkE activity (Fig 2C, S2A and S2B Fig). This was particularly marked at E14.5, with this population yielding 20% CD41^+^ megakaryocytes following OP9 co-culture, compared to <5% in E12.5 and E13.5 cultures (Fig 2D and 2E, S2C–S2F Fig).

To achieve a more accurate estimate of the potentiality of *P2-hCD4*^*-*^ and *P2-hCD4*^*+*^ CD41^-^ CD150^+^ MEPs, we performed single cell OP9 co-culture assays (Fig 2F). Single *P2-hCD4*^*-*^ and *P2-hCD4*^*+*^ CD41^-^ CD150^+^ MEPs displayed plating efficiencies of 33.3% and 28.5% respectively in these conditions. Alternatively, replating in MethoCult yielded 60.4% and 51.0% plating efficiencies for *P2-hCD4*^*-*^ and *P2-hCD4*^*+*^ CD41^-^ CD150^+^ MEPs respectively (Fig 2G). Under both culture conditions, approximately 15% of positive wells derived from *P2-hCD4*^*+*^ CD41^-^ CD150^+^ MEPs yielded both megakaryocytic and erythroid cells. By contrast, no single *P2-hCD4*^*-*^ CD41^-^ CD150^+^ MEPs demonstrated dual lineage specification. We therefore established that MEP bipotentiality is restricted to the CD41^-^ CD150^+^ *P2-hCD4*^*+*^ MEP subfraction, comprising approximately 1% of immunophenotypic MEPs and fewer than 0.05% of total live cells in E14.5 fetal liver (Fig 2A and 2B).

To fully demonstrate the increased megakaryocytic lineage commitment in E14.5 fetal liver *P2-hCD4*^*+*^ CD41^-^ CD150^+^ MEPs compared to their *P2-hCD4*^*-*^ counterparts, we cultured both cell types in pro-myeloid medium for 14 hours (Fig 2H and 2I). *P2-hCD4*^*+*^ CD41^-^ CD150^+^ MEPs produced more MkPs than the *P2-hCD4*^*-*^ population, which yielded greater proportions of erythroid-dominated CD150^-^ MEPs. We therefore demonstrated that the *P2-hCD4*^*+*^ fraction represents a pro-megakaryocyte subpopulation of immunophenotypic MEPs in E14.5 FL.

### *Runx1-P2-hCD4*^*+*^ CMPs have enriched multilineage output

**Fig 3:**
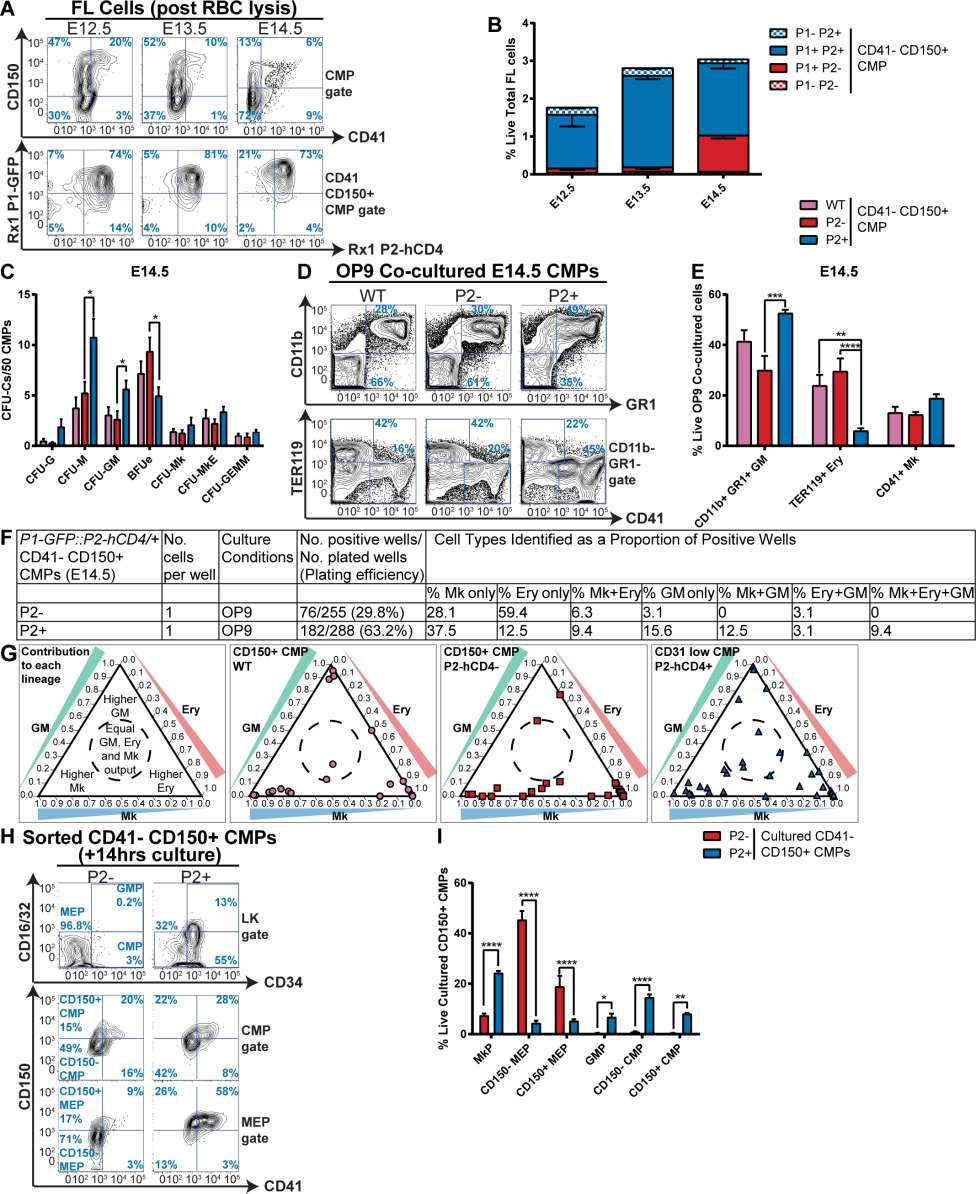
*Runx1-P2-hCD4*^*+*^ CMPs have enriched multilineage output. **A-B.** Expression of *Runx1-P1-GFP* and *P2-hCD4* in *P1-GFP*::*P2-hCD4/+* E12.5, E13.5 and E14.5 FL CD41^-^ CD150^+^ CMPs. A. Representative FACS plots. B. Quantitation of the proportions of *P1*^*-*^ *P2*^*-*^, *P1*^*+*^ *P2*^*-*^, *P1*^*+*^ *P2*^*+*^ and *P1*^*-*^ *P2*^*+*^ CMPs as a percentage of total live red blood cell lysed fetal liver cells. N = 3 **C.** Differential CFU-C activity of wild type, *P2-hCD4*^*-*^ and *P2-hCD4*^*+*^ E14.5 FL CD41^-^ CD150^+^ CMPs. N = 7 **D-E.** Lineage output of day 7 OP9 co-cultured wild type, *P2-hCD4*^*-*^ and *P2-hCD4*^*+*^ CD41^-^ CD150^+^ CMPs. D. Representative FACS plots of CD11b, GR1, TER119 and CD41. E. Proportion of granulocyte/monocyte, erythroid and megakaryocyte cells. N = 7 **F-G.** Lineage output of single cultured *P2*^*-*^ and *P2*^*+*^ CMPs in OP9 co-cultures. F. Table of plating efficiency. G. Ternary plots displaying proportions of granulocyte/monocyte, erythroid and megakaryocyte cells in each positive well for wild type, *P2-hCD4*^*-*^ and *P2-hCD4*^*+*^ CD41^-^ CD150^+^ CMPs. **H-I.** Short-term (14 hours) differentiation of *P2*^*-*^ and *P2*^*+*^ CD41^-^ CD150^+^ CMPs in pro-myeloid liquid culture. H. Representative FACS plots of CD16/32/CD34 (top) and CD150/CD41 (middle and bottom) expression in the LK gate of cultured CMPs. I. Proportions of immunophenotypic MkPs, CD41^-^ CD150^-^ MEPs, CD41^-^ CD150^+^ MEPs, GMPs, CD41^-^ CD150^-^ CMPs and CD41^-^ CD150^+^ CMPs in short-term cultures. N = 3.

Having established that *Runx1-P2-hCD4* expression heterogeneity in CD41^-^ CD150^+^ MEPs is associated with different lineages, we turned to CD41^-^ CD150^+^ CMPs. E12.5, E13.5 and E14.5 fetal liver CD41^-^ CD150^+^ CMPs mostly express *Runx1-P1-GFP* (81%, 86% and 94% respectively, Fig 3A and 3B). Additionally, most CD150^+^ CMPs co-express *P2-hCD4* (74% in E12.5, 81% in E13.5 and 73% in E14.5 fetal liver). At all three stages, colony-forming output was skewed in favor of CFU-Ms and away from BFUes in *P2-hCD4*^*+*^ CMPs compared to *P2-hCD4*^*-*^ CMPs (Fig 3C, S3A and S3B Fig). Moreover, CFU-GM output was enriched at E13.5 and E14.5, and multipotent CFU-GEMM activity was enriched in *P2-hCD4*^*+*^ CMPs at E13.5 compared to the *P2-hCD4*^*-*^ populations (Fig 3C, S3B Fig).

OP9 co-culture of the bulk *P2-hCD4*^*-*^ and *P2-hCD4*^*+*^ CD41^-^ CD150^+^ CMP populations revealed changing lineage output in E12.5, E13.5 and E14.5 fetal liver (Fig 3D and 3E, S3C–S3F Fig). Notably, pro-GM/anti-Erythroid bias was evident in E14.5 *P2-hCD4*^*+*^ CMPs. Analysis of single co-cultured CMPs (Fig 3F and 3G) confirmed this, as almost 60% of E14.5 *P2-hCD4*^*-*^ CMPs produced solely erythroid cells, compared to 12.5% of *P2-hCD4*^*+*^ CMPs. The granulocyte/monocyte-restricted fraction (“GM only”) represented 3% and 16% of the *P2-hCD4*^*-*^ and *P2-hCD4*^*+*^ CMPs respectively. Megakaryocytic output was similarly enhanced in *P2-hCD4*^*+*^ CMPs, with 38% possessing Mk-restricted and 9% demonstrating bipotential megakaryocytic/erythroid (Mk+Ery) activity, (compared to 28% and 6% of *P2-hCD4*^*-*^ CMPs). Importantly, we observed multipotent (balanced Mk+Ery+GM) lineage output in *P2-hCD4*^*+*^ CMPs only, albeit in <10% of positive wells. This suggests multilineage activity is highly restricted to *Runx1*-*P2-hCD4*^*+*^ CMPs in E14.5 fetal liver.

Following culture in pro-myeloid medium for 14 hours, the majority of *P2-hCD4*^*-*^ CMPs had acquired a CD34^-^ MEP phenotype (90% of total cells, Fig 3H and 3I). Of these, 71% had downregulated CD150 expression, suggesting erythroid commitment. By contrast, >20% of cultured *P2-hCD4*^*+*^ CMPs had acquired an MkP immunophenotype (upregulating CD41), 10% had a CD41^-^ CD150^-^ MEP immunophenotype, and 10% expressed CD16/32, thus acquiring a GMP immunophenotype. *Runx1-P2-hCD4*^*+*^ CMPs can therefore efficiently produce immunophenotypic MkPs, MEPs and GMPs, whereas *P2-hCD4*^*-*^ CMPs appear committed to the generation of immunophenotypic MEPs.

### CD31 expression distinguishes multilineage HPCs from more restricted progenitors

Having established that *Runx1*-*P2-hCD4*^*+*^ MEPs and CMPs are enriched in multilineage progenitors, we endeavored to identify cell surface markers which correlated with *P2-hCD4* expression in E14.5 fetal liver. The aim was to achieve the prospective isolation of these populations from wild type (WT) or other transgenic mouse lines in the absence of the *Runx1*-*P2-hCD4* reporter. Firstly, we performed Single Cell RNA Sequencing on CD41^-^ CD150^+^ *P2-hCD4*^*-*^ and *P2-hCD4*^*+*^ MEPs and CMPs. By performing a principal component analysis, we observed a clear transition in transcriptomic activity from the P2- MEP to the P2+ CMP (S4A Fig). In particular, erythroid gene expression (for example, *Klf1*) was highly upregulated in the P2- MEPs being downregulated with the upregulation of *P2-hCD4* and also sharing an inverse relationship with the megakaryocytic transcription factor *Fli1* and the granulocyte/monocyte transcription factor *Spi1* (*Pu*.*1*). (Full lists of differentially expressed genes between P2-/+ MEPs and P2-/+ CMPs can be found in S5 Table and S6 Table respectively.)

In order to identify markers which may aid the isolation of *P2-hCD4*^*-*^ and *P2-hCD4*^*+*^ MEPs, we analyzed the expression of various cell surface markers, the aim being to identify genes which are upregulated or downregulated in some or all *P2-hCD4*^*+*^ MEPs compared to *P2-hCD4*^*-*^ MEPs. Promising candidates included *Cd48*, *Pecam1* (*Cd31*), *Ptprc* (*Cd45*), *Eng* (*Endoglin*), *Itgb1* (*Cd29*) and *Tek* (*Tie2*) (S4B Fig). We therefore screened antibodies which had been raised against these markers and other heterogeneously-expressed markers in fetal liver (S4C Fig).

**Fig 4:**
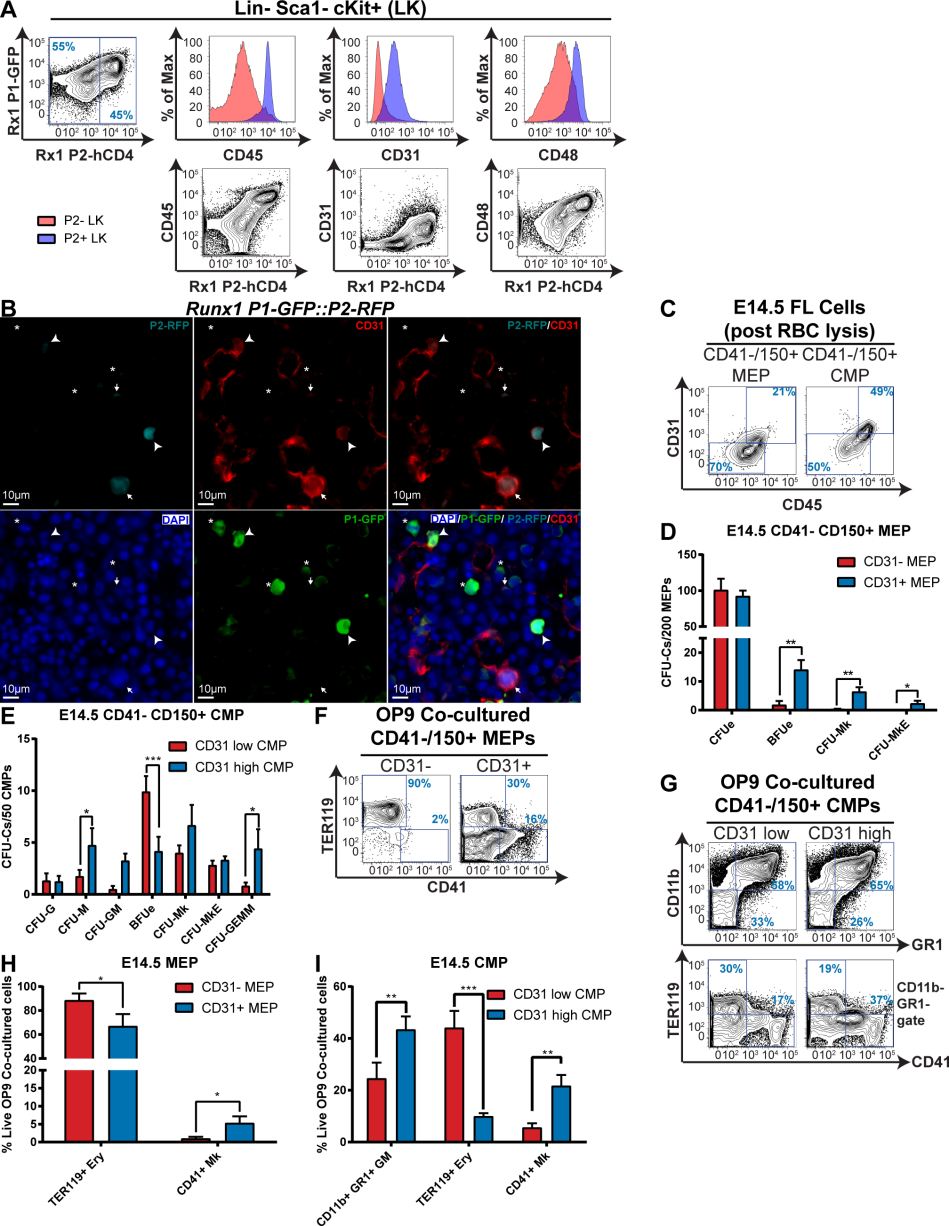
CD31 expression distinguishes multilineage HPCs from more restricted progenitors. **A.** Representative FACS plots of CD45, CD31 and CD48 expression in *P1-GFP*::*P2-hCD4/+* E14.5 FL *P2-hCD4*^*-*^ and *P2-hCD4*^*+*^ LK cells. **B.** Representative micrographs of DAPI, P2-RFP, CD31 and P1-GFP Immunofluorescence staining in *Runx1-P1-GFP*::*P2-RFP* E14.5 fetal liver. Examples of *P1-GFP*^*-*^ *P2-RFP*^*+*^ (white arrows), *P1-GFP*^*+*^ *P2-RFP*^*+*^ (white arrowheads) and *P1-GFP*^*+*^ *P2-RFP*^*-*^ (white asterisks) cells are indicated. **C.** Representative FACS plots of CD31/CD45 expression in wild type E14.5 fetal liver CD41^-^ CD150^+^ MEPs and CMPs. **D-E.** Differential CFU-C activity in wild type E14.5 fetal liver CD31^-/+^ MEPs (D, N = 7) and CD31^low/high^ CMPs (E, N = 6). **F-I.** Lineage output of OP9 co-cultured MEPs and CMPs. F. Representative FACS plots of TER119 and CD41 expression of day 7 OP9 co-cultured MEPs. G. Representative FACS plots of CD11b, GR1, TER119 and CD41 expression of day 7 OP9 co-cultured CMPs. H. Proportion of TER119^+^ erythroid cells and CD41^+^ megakaryocyte cells in day 7 OP9 MEP co-cultures. I. Proportion of granulocytes/monocytes, erythroid cells and megakaryocytes in day 7 OP9 CMPs co-cultures. N = 8.

Promising candidates included the leukocyte common antigen, protein tyrosine phosphatase receptor type C (PTPRC) or CD45; CD48 antigen; and platelet/endothelial cell adhesion molecule 1 (PECAM1) or CD31. All three markers correlated positively with *Runx1-P2-hCD4* in LK hematopoietic progenitors (Fig 4A). Immunofluorescence staining, performed on total fetal liver sections from *P1-GFP*::*P2-RFP* E14.5 embryos, demonstrated that although *P2-RFP* cells are comparatively rare (2.89% of total DAPI^+^ cells, Fig 4B, S4D Fig) in E14.5 FL, a large proportion (64.4%) have cell surface CD31 staining. We therefore proceeded to characterize CD31 expression in CD41^-^ CD150^+^ MEPs and CMPs.

In wild type fetal liver, 20% of CD41^-^ CD150^+^ MEPs expressed CD31 (Fig 4C). By comparison, >90% of CD41^-^ CD150^+^ CMPs were CD31^+^, although CD31^low^ and CD31^high^ populations were discernible. We therefore separated CD41^-^ CD150^+^ CMPs into the 50% lowest and 50% highest CD31-expressing subfractions (CD31^low^ and CD31^high^ CMPs respectively) for further characterization.

All MEPs and CMPs expressed CD45 and CD48 (Fig 4C, S4E Fig). Expression of the RUNX1 target genes *Gfi1b* and *Gfi1* in MEPs and CMPs was also assessed, utilizing the *Gfi1b-GFP* and *Gfi1-GFP* reporter mouse lines (S4F and S4G Fig). The megakaryocytic/erythroid transcription factor *Gfi1b* was expressed in all CD41^-^ CD150^+^ MEPs and CMPs, although the *Gfi1b-GFP* median fluorescence intensity (MFI) was decreased in some CD31^high^ CMPs (S4F Fig). In contrast, granulocyte/monocyte-associated *Gfi1-GFP* was expressed in only 8% of CD41^-^ CD150^+^ MEPs and 39% of CD41^-^ CD150^+^ CMPs, the highest *Gfi1-GFP* MFI being associated with high CD31 expression (S4G Fig).

Assessing the CFU-C activity of bulk and single MEP cells in MethoCult culture (Fig 4D, S4H Fig) revealed that all CFU-Mk and CFU-MkE activity resided with CD31^+^ MEPs; CD31^-^ MEPs possessed CFUe and BFUe activity only. OP9 co-culture confirmed this lineage output (Fig 4F and 4H; S4I Fig), as CD41^+^ megakaryocyte cells were absent in CD31^-^ MEP co-cultures but present in 7% of positive wells derived from CD31^+^ MEPs (S4I Fig). This suggested that CD31^+^ MEPs possess some Mk lineage specificity (albeit in only 7–20% of cells), whereas CD31^-^ MEPs solely constitute erythroid progenitors.

Erythroid lineage output was enriched in CD31^low^ CMPs compared to CD31^high^ CMPs (Fig 4E, 4G and 4I; S4J Fig); 20% of CD31^low^ CMPs produced BFUes and 54% of single co-cultured cells yielded solely TER119^+^ erythroid cells. CD11b^+^ Gr1^+^ granulocyte/monocyte and CD41^+^ megakaryocyte cell output was enhanced considerably in CD31^high^ CMPs. Interestingly, although CFU-GEMM activity was enriched in CD31^high^ CMPs, the proportion of positive co-cultured wells yielding megakaryocyte/erythroid/granulocyte/monocyte cells was higher for CD31^low^ CMPs. This was probably due to the overall plating efficiencies being higher for CD31^high^ CMPs. Therefore, both CD31^low^ CMPs and CD31^high^ CMP populations are heterogeneous, albeit skewed towards erythroid and GM/megakaryocytic specification respectively.

The short-term culture of these populations revealed hierarchical relationships; CD31^+^ MEPs gave rise to CD31^-^ MEPs and MkPs, whereas CD31^-^ MEPs rapidly downregulated CD150 (S4K and S4M Fig). The CD31^low^ CMPs are skewed to a pro-MEP fate, whereas CD31^high^ CMPs efficiently produce GMPs and CD31^+^ MEPs (S4L and S4N Fig). CD31^low^ CMPs therefore comprise a more advanced pro-erythroid fraction, but can also produce CD31^high^ CMPs, suggesting some granulocyte/monocyte commitment.

As previously indicated, the provenance of the fetal liver myeloid progenitors is not entirely clear. Following initial fetal liver colonization at E11.5, the HSCs expand exponentially and the numbers of repopulating units (per embryo equivalent) peak by E16, as fetal bone marrow colonization is underway (having begun from E15) [29,30,44]. To add weight to the hypothesis that the CD150^+^ MEP and CMP populations are fetal liver HSC-derived, we analyzed these fractions in E16.5 fetal liver. Firstly, upon analyzing the LK hematopoietic progenitor compartment of the *Runx1 P1-GFP*::*P2-hCD4* E16.5 fetal liver, we observed that the frequencies of *P2*^*+/-*^ CD41^-^ CD150^+^ MEPs and CMPs closely resembled that observed in E14.5 fetal liver (S5A and S5B Fig). Upon isolating these populations, we observed that the *P2*^*+*^ CD41^-^ CD150^+^ MEPs and CMPs displayed decreased erythroid and increased megakaryocytic/GM output compared to the *P2*^*-*^ fractions (S5C–S5H Fig). We also confirmed that *Runx1 P2-hCD4* expression correlated well with CD31 expression in the E16.5 fetal liver LK fractions (S5I-J) and that wild type E16.5 CD31^+/-^ MEPs and CD31^low/high^ CMPs displayed similar lineage specificities, particular concerning erythroid output, compared to their *P2*^*+/-*^ equivalents (S5K-P). This therefore suggests that similar MEP and CMP populations can be discerned in E16.5 fetal liver as in E14.5 fetal liver and therefore that the hematopoietic hierarchy is maintained 5 days after fetal liver colonization, even after the shift to bone marrow colonization has begun.

### *Runx1*-null CD31^low^ CMPs have impaired erythroid cell production

Defining restricted fetal liver hematopoietic progenitor cell compartments should provide the ability to identify the impact of genetic alterations with greater precision. For example, *Runx1-flox*::*Vav1-Cre* conditional knockout mice display impaired fetal liver erythroid and megakaryocytic maturation (S6A–S6H Fig).

The absence of RUNX1 protein in *Runx1-flox/flox*::*Vav1-Cre* (*Runx1-del/del*, S6A Fig) apparently caused a block in the upregulation of TER119 expression. Consequently, there was an accumulation of the immature S0-S2 erythroid lineage subsets, but a decrease in the CD71^high^ TER119^high^ S3 population (S6B-E) [45]. Following megakaryocytic culture, *Runx1-del/del* fetal liver samples produced more CD41^high^ megakaryocytes than their WT and *Runx1-del/+* littermates, but the majority of these did not upregulate CD42d, reflecting the previously described megakaryocytic maturation block (S6F and S6G Fig). This was confirmed by the complete absence of morphologically mature megakaryocytes, following staining with May-Grünwald Giemsa reagent (S6H Fig). This megakaryocyte maturation block is reminiscent of Familial Platelet Disorder with Predisposition to Acute Myeloid Leukemia (FPD/AML), almost exclusively the result of germline heterozygous *RUNX1* gene mutations/deletions [46,47].

**Fig 5:**
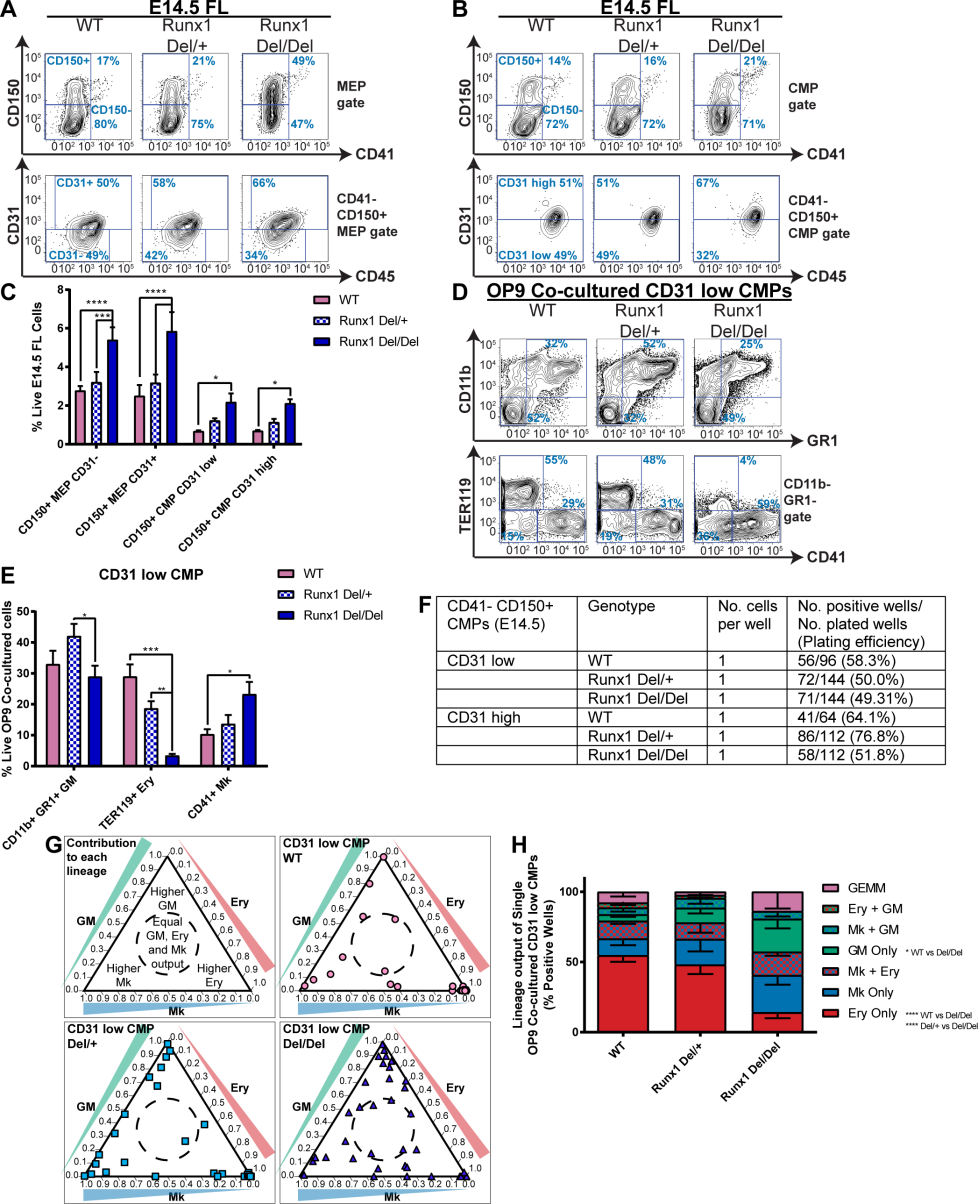
*Runx1-*null CD31^low^ CMPs have impaired erythroid cell production. **A-C.** Identifying CD31-expressing CD41^-^ CD150^+^ MEPs and CMPs in *Runx1* wild type, heterozygous (Del/+) and null (Del/Del) E14.5 fetal liver. Representative FACS plots of CD150/CD41 and CD31/CD45 expression in MEPs (A) and CMPs (B). C. Quantitation of CD31^-/+^ MEPs and CD31^low/high^ CMPs. N = 6 **D-E.** Lineage output of OP9 co-cultured *Runx1* wild type, heterozygous and null CD31^low^ CMPs. D. Representative FACS plots of CD11b, GR1, TER119 and CD41 expression of day 7 OP9 co-cultured CD31^low^ CMPs. E. Proportion of granulocyte/monocyte, erythroid and megakaryocyte cells in day 7 OP9/CD31^low^ CMPs co-cultures. N = 4 **F-H.** Lineage output of single OP9 co-cultured *Runx1* wild type, heterozygous and null CD31^low^ and CD31^high^ CMPs. F. Plating efficiency of CD31^low^ and CD31^high^ CMPs. G. Ternary plots displaying proportions of granulocyte/monocyte, erythroid and megakaryocyte cells in each positive well for CD31^low^ CMPs. H. Proportions of unilineage (Ery, Mk and GM only) and multilineage (Mk+Ery, Mk+GM, Ery+GM and GEMM) wells derived from CD31^low^ CMPs. N = 4.

To further investigate the impact of the absence of RUNX1, we performed a detailed characterization of the hematopoietic progenitor composition of *Runx1-del/del* E14.5 fetal liver using our CD31 staining protocol and compared it to wild type and *Runx1-del/+* heterozygous littermates (Fig 5A–5C, S7A and S7B Fig). CD41^-^ CD150^+^ CD31^+/-^ MEP and CD31^low/high^ CMP populations were all expanded in *Runx1-del/del* fetal liver, as was the pro-GM CD41^-^ CD150^-^ CMP fraction (Fig 5B–5C, S7B Fig). By contrast, pro-erythroid CD41^-^ CD150^-^ MEPs were depleted (Fig 5A, S7B Fig), suggesting the absence of RUNX1 may block erythroid differentiation during fetal liver hematopoiesis.

Assessment of CFU-C activities (S7C–S7F Fig) uncovered a modest increase in CFUe production by *Runx1-del/del* CD41^-^ CD150^+^ CD31^+^ MEPs but decreased CFU-MkE and CFU-GEMM activity in *Runx1-del/del* CD41^-^ CD150^+^ CD31^high^ CMPs. Total CFU-Mk output did not appear to be decreased, but defective megakaryocytic maturation was evident as morphologically mature CFU-Mks were absent from *Runx1-del/del* hematopoietic progenitor cultures (S7E and S7F Fig).

Bulk OP9 co-culture revealed decreased mature TER119^+^ erythroid cell production by *Runx1-del/del* CD31^low^ CMPs and CD31^-/+^ MEPs, but not CD31^high^ CMPs (Fig 5D and 5E, S7G and S7K Fig), confirming an erythroid maturation block. *Runx1-del/del* CD31^low^ CMPs had increased CD41^+^ megakaryocytic output, in line with the previously reported increased proliferation/reduced maturation in this lineage [48]. However, this did not answer the question of whether lineage specification was altered by the absence of RUNX1 causing a perturbation of the numbers of pro-erythroid, pro-megakaryocyte and/or pro-granulocyte/monocyte progenitors in hematopoietic progenitor pools.

To address this question, we performed single cell OP9 co-cultures and observed similar total plating efficiencies for wild type, *Runx1-del/+* and *Runx1-del/del* populations (Fig 5F). Whereas >50% of wild type and *Runx1-del/+* CD31^low^ CMPs solely produced TER119^+^ Erythroid cells, this was reduced 4-fold in *Runx1-del/del* CD31^low^ CMPs (Fig 5G and 5H, S7L Fig), with a concurrent increase in pro-GM progenitors. Erythroid cells were more modestly reduced in CD31^high^ CMP cultures (S7M–S7O Fig), but this was not very impactful as the population has a lower pro-erythroid pool in wild type fetal liver compared to its CD31^low^ counterpart. Moreover, the Median Fluorescent Intensity of the TER119^+^ erythroid cells was decreased in *Runx1-del/del* CD31^low^ CMP cultures compared to that of wild type littermates (S7P Fig). This confirmed that the proportion of pro-erythroid progenitors residing in the fetal liver CD31^low^ CMP fraction is diminished in *Runx1*-null E14.5 embryos, and that differentiation of these progenitors is impaired.

A key advantage of identifying these highly purified CD31^low^ and CD31^high^ CMP fractions in *Runx1* null fetal liver is the ability to analyze underlying cell-intrinsic changes driving the lineage specification and maturation defects with greater precision, particularly at a transcriptome level. We therefore analyzed the expression of key HSC and lineage-associated transcriptional regulators in wild type, *Runx1-del/+* and *Runx1-del/del* CD31^low^ and CD31^high^ CMPs (S7Q Fig). One key observation was that several key HSC and megakaryocyte/erythrocyte-associated transcription factors (*Tal1*, *Gfi1b* and *Klf1*) were downregulated in wild type CD31^high^ CMPs compared to wild type CD31^low^ CMPs, reflecting the megakaryocytic/erythroid lineage commitment which accompanies CD31 downregulation in the CMP compartment and demonstrating that CD31^low^ and CD31^high^ CMPs represent transcriptionally distinct progenitor subsets.

Notably, expression of *Tal1*, *Gfi1b* and *Klf1* did not differ between wild type, *Runx1-del/+* and *Runx1-del/del* CD31^high^ CMPs. However, they were substantially downregulated in *Runx1-del/del* CD31^low^ CMPs compared to their wild type and *Runx1-del/+* equivalents. In fact, the transcription factors *Tal1*, *Gfi1b*, *Klf1* and *Gata2*, plus the megakaryocytic/erythroid lineage markers *Itga2b*, *Pf4* and *Epor* (S7R Fig), were all expressed at comparable levels in *Runx1-del/del* CD31^low^ CMPs to CD31^high^ CMPs (of all genotypes). This supports the hypothesis that the absence of RUNX1 results in a differentiation block between the CD31^high^ and CD31^low^ CMPs. Additionally, it indicates that the megakaryocytic/erythroid maturation defects are established even at this early stage, with a failure to upregulate key maturation-associated transcripts.

Interestingly, unlike the pro-erythroid transcription factor *Klf1*, the pro-megakaryocytic *Fli1* and the pro-GM *Spi1* and *Gfi1* were not significantly different in CD31^low^ and CD31^high^ CMPs; nor were they impacted by the absence of RUNX1. This may indicate that commitment to the erythroid lineage is the default position in the FL CD31^high^-to-CD31^low^ CMP transition, which was hindered in the absence of RUNX1. We therefore clearly demonstrate the benefits of isolating highly purified myeloid progenitors to aid in the understanding of the mechanistic basis of congenital hematopoietic defects in the fetal liver.

### *Runx1-P1-MRIPV* CD31^high^ CMPs have impaired Mk specification

We recently reported that deleting the RUNX1C isoform in adult mice, whilst maintaining total RUNX1 expression, resulted in mild thrombocytopenia, due to impaired megakaryocytic specification but with normal megakaryocyte maturation. To achieve this we utilized the *Runx1-P1-MRIPV* line, in which the *P1-*encoded RUNX1C isoform is replaced by the *P2-*encoded RUNX1B isoform [39]. Given the contrast between this phenotype and that of the *Runx1-del/del* adult model, we decided to interrogate *P1-MRIPV* fetal liver hematopoiesis.

Having confirmed *P1-MRIPV/MRIPV* and *P1-MRIPV/+* fetal liver cells maintain RUNX1 protein expression at wild type levels (S8A Fig), we examined erythroid and megakaryocytic maturation in these samples (S8B–S8G Fig). Unlike in *Runx1-*null fetal liver, *P1-MRIPV/*MRIPV fetal liver displayed only a modest increase in the CD71^-/low^ TER119^-^ S0 fraction and corresponding decrease in the CD71^high^ TER119^high^ S3 fraction. This suggested that the absence of RUNX1C/overexpression of RUNX1B does not significantly impact erythroid maturation (S8B–S8E Fig). Following megakaryocyte culture, we observed that megakaryocytic maturation was also unimpaired, as the proportions of mature CD41^high^ CD42d^+^ megakaryocytes were similar in wild type, *P1-MRIPV/+* and *P1-MRIPV/MRIPV* cultures (S8F and S8G Fig).

**Fig 6:**
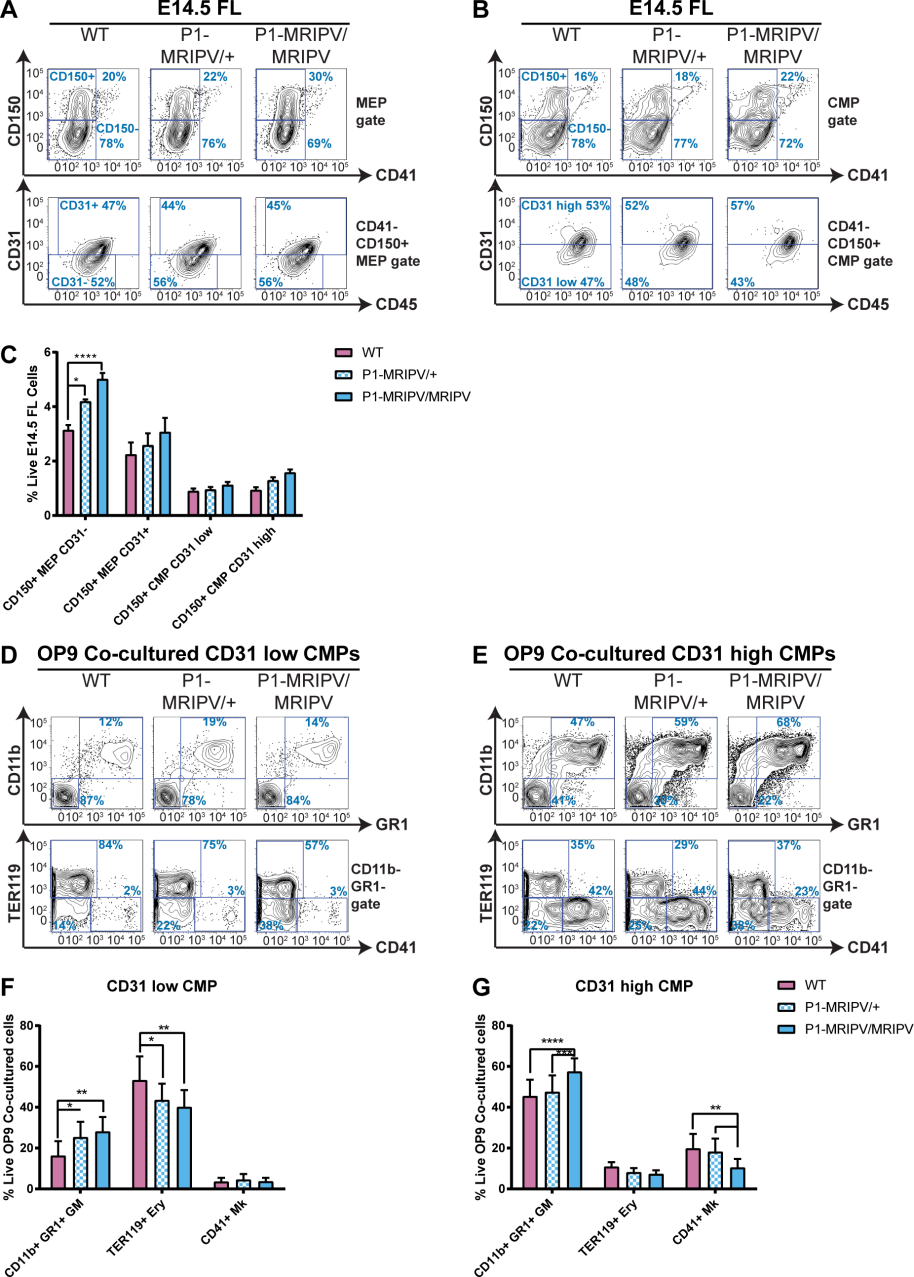
*Runx1-P1-MRIPV* CD31^high^ CMPs have impaired Mk specification. **A-C.** CD31-expressing CD41^-^ CD150^+^ MEPs and CMPs in *Runx1* wild type, *P1-MRIPV/+* and *P1-MRIPV/MRIPV* E14.5 fetal liver. Representative FACS plots of CD150/CD41 and CD31/CD45 expression in MEPs (A) and CMPs (B). C. Quantitation of CD31^-/+^ MEPs and CD31^low/high^ CMPs. N = 7 **D-G.** Lineage output of OP9 co-cultured *Runx1* wild type, *P1-MRIPV/+* and *P1-MRIPV/MRIPV* CD31^low^ and CD31^high^ CMPs. D-E. Representative FACS plots of CD11b/GR1 and TER119/CD41 expression of day 7 OP9 co-cultured CD31^low^ (D) and CD31^high^ CMPs (E). F-G. Proportions of granulocyte/monocyte, erythroid and megakaryocytic cells in day 7 OP9 co-cultured CD31^low^ (F) and CD31^high^ (G) CMPs. N = 5.

We next proceeded to characterize fetal liver myeloid hematopoietic progenitor compartments using our CD31 staining protocol (Fig 6A–6C, S9A and S9B Fig). The impact of deleting RUNX1C alone was far more restricted, the CD41^-^ CD150^+^ CD31^-^ MEP being the only expanded population identified in *P1-MRIPV/MRIPV* fetal liver (Fig 6A–6C). As the CD31^-^ MEP is highly erythroid-biased, we investigated whether *P1-MRIPV/MRIPV* fetal liver progenitors displayed pro-erythroid/anti-megakaryocytic bias. CFU-C assays revealed increased CFUe activity in the *P1-MRIPV/MRIPV* CD31^+^ MEP, but decreased CFU-Mk and increased CFU-M activity in the CD31^high^ CMP (S9C–S9F Fig).

OP9 co-culture suggested the absence of RUNX1C did not impair the lineage output of CD31^-^ or CD31^+^ MEPs (S9G–S9I Fig). Whilst the *P1-MRIPV/MRIPV* CD31^low^ CMP was modestly affected, substantially decreased CD41^+^ megakaryocyte output and increased CD11b^+^ GR1^+^ granulocyte/monocyte output were observed for *P1-MRIPV/MRIPV* CD31^high^ CMPs (Fig 6D–6G). We therefore hypothesized that the dominant impact of deleting RUNX1C (or overexpressing RUNX1B) on megakaryocytic versus granulocyte/monocyte specification occurs in the CD31^high^ CMP fraction.

This hypothesis was confirmed through short-term culture of the CMP populations (S9J–S9M Fig), as *P1-MRIPV/MRIPV* CD31^high^ CMPs produced fewer MkPs and more GMPs than their wild type counterparts, but the lineage specification of CD31^low^ CMPs was largely unaffected. Therefore, in contrast to the impaired erythroid specification of *Runx1-del/del* CD31^low^ CMPs, *P1-MRIPV/MRIPV* CD31^high^ CMPs display impaired megakaryocytic specification. This demonstrates that the RUNX1B and RUNX1C isoforms have distinct roles during fetal liver hematopoiesis, akin to adult bone marrow hematopoiesis, which can be uncovered using our enhanced fetal liver HPC purification strategies.

## Discussion

**Fig 7:**
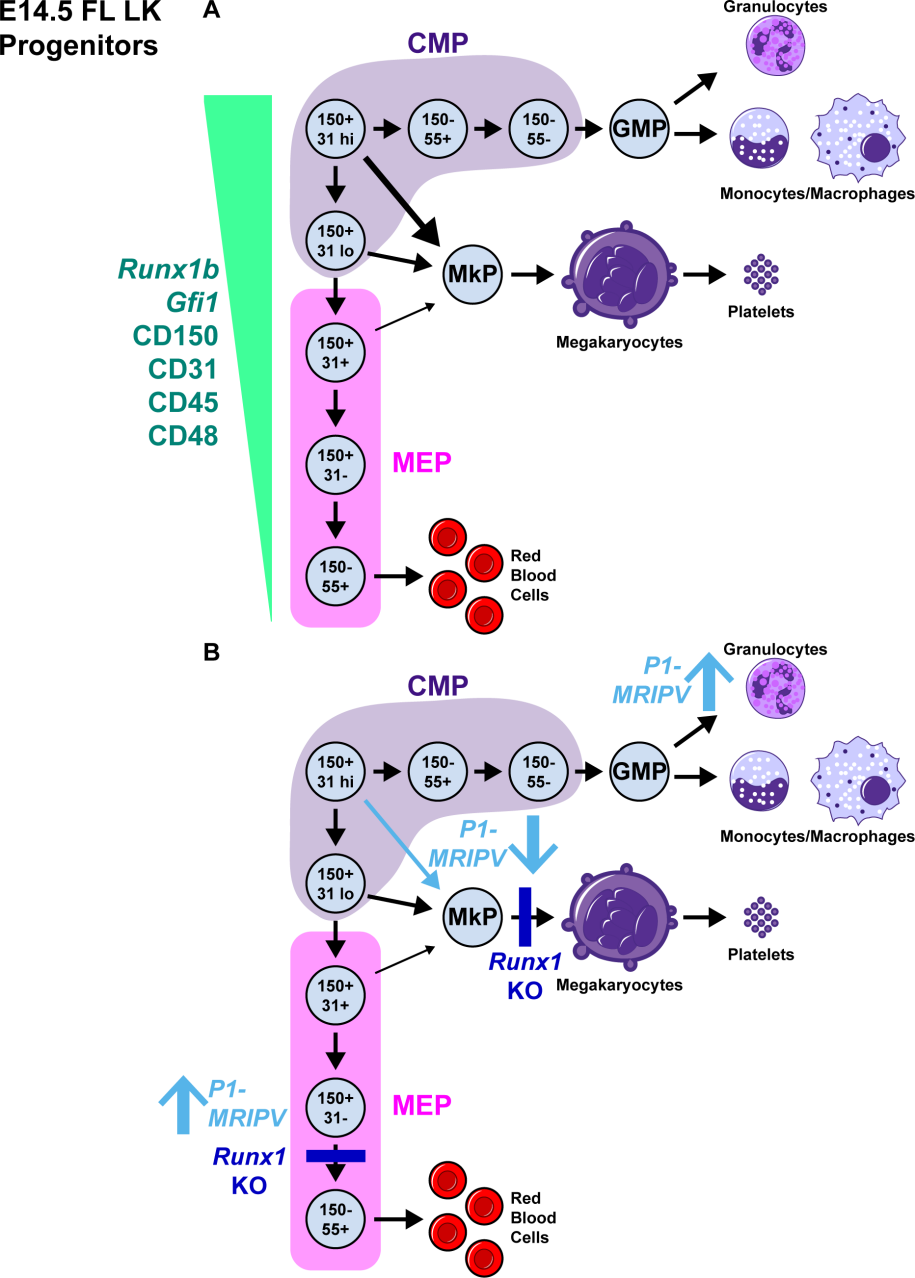
Distinct subsets within the classically defined CMP and MEP compartments can be segregated on the basis of CD55, CD150 and CD31 expression. **A-B.** Proposed models of fetal liver myeloid progenitor hierarchy. A. In wild type fetal liver, commitment to the erythroid lineage correlates with downregulation of the *Runx1b* and *Gfi1* TFs, plus the cell surface markers CD150, CD31, CD45 and CD48. B. RUNX1-null fetal liver hematopoietic progenitors have impaired Mk and erythroid differentiation (dark blue bars), whereas RUNX1C-null (*P1-MRIPV)* hematopoietic progenitors have impaired megakaryocyte specification, displaying enhanced erythroid and granulocyte/monocyte commitment (light blue arrows).

Bone marrow myeloid hematopoietic progenitor cells have been extensively characterized, including the recent demonstration that immunophenotypically similar CMPs can be separated into distinct PU.1-eYGP^high^ GATA1-mCherry^-^ pro-granulocyte/monocyte and PU.1-eYFP^low^ GATA1-mCherry^+^ pro-megakaryocyte/erythroid fractions [40]. This therefore confirms that lineage fate decisions had commenced upstream of the CMPs in ancestral HSPCs. By comparison, the delineation of myeloid lineage-restricted hematopoietic progenitors in fetal liver lags critically behind. We therefore attempted to further compartmentalize the immunophenotypic MEP and CMP fractions to provide a more detailed hematopoietic hierarchy (summarized in Fig 7A). CD55 and CD150 were obvious lead candidates, as CD55 was successfully utilized by Guo *et al* to subdivide adult CMPs into pro-megakaryocyte/erythroid and pro-granulocyte/monocyte fractions [41]; additionally, CD150 is a well-established pro-megakaryocytic/erythroid and HSC marker [5,32,43,49,50]. We observed that the entirety of megakaryocytic/erythroid bipotential and granulocyte/monocyte/megakaryocyte/erythrocyte multipotential CFU-C output resides respectively in the CD55^+^ CD150^+^ MEP and CD55^+^ CD150^+^ CMP fetal liver fractions. However, the CFU-Mk and CFU-MkE output of CD55^+^ CD150^+^ MEPs remained low (~5%), highlighting the need for further refined hematopoietic progenitor subfractionation.

For this, we identified distinct myeloid hematopoietic progenitor subsets on the basis of *P2-hCD4* expression in our *Runx1 P1-GFP*::*P2-hCD4* reporter mouse. To broaden the application of this finding, and not rely on the *P1-GFP*::*P2-hCD4* reporter mouse, we searched for cell surface markers which correlated with *P2-hCD4* expression. Following a screen of markers associated with heterogeneous fetal liver expression or multipotency/lineage specification in bone marrow hematopoietic progenitors [51], we identified CD31, CD45 and CD48 as strong candidates. Downregulation of the pan-leukocyte marker, CD45 [52,53], and the bone marrow hematopoietic progenitor, lymphocyte and macrophage-associated CD48 [49,54–56] upon fetal liver erythroid commitment concurs with adult bone marrow erythroid progenitor specification [51]. CD31, meanwhile, is expressed in endothelial progenitor cells and their mature progeny [57–60]. CD31-null mice are viable and do not exhibit obvious vascular defects; nonetheless CD31 plays significant roles in vascular remodeling and tumor metastatic progression as well as in adhesion, survival, migration and activation of hematopoietic cells [61–70]. In E14.5 fetal liver, CD31 is predominantly expressed in cells lining the hepatic vessels, but also in some *Runx1*^*+*^ hematopoietic stem and progenitor cells. We observed CD31 expression was highly enriched in the fetal liver myeloid progenitors with the greatest granulocyte/monocyte and megakaryocytic output. This concurs somewhat with the observation that in fetal liver and bone marrow, the entire multilineage LSK hematopoietic stem and progenitor cell fraction expresses CD31 [57,71,72]. Contrastingly, the bone marrow LK CD31^+^ fraction was deficient in granulocyte/monocyte output, possessing chiefly short-term erythroid repopulating cells, whereas we demonstrate here that erythroid lineage commitment in fetal liver coincides with CD31 downregulation. This suggests CD31-expressing short-term progenitors are not equivalent throughout mouse ontogeny. The shift from CD31^+^ pro-GM/megakaryocytic fetal liver hematopoietic progenitors to bone marrow erythroid progenitors may reflect distinct interactions with their respective niches. CD31-null adult mice have more steady state circulating progenitors, as hematopoietic progenitors fail to migrate across the bone marrow vasculature [68]. This phenotype was observed whether CD31 was deleted in hematopoietic or endothelial cells or both. The retention of the numerous, highly clonogenic CD31^-^ fetal liver erythroid progenitors in their niche may be less crucial than for CD31^+^ bone marrow erythroid progenitors, which are replaced less frequently by more quiescent precursors.

High CD31 expression in fetal liver pro-GM/megakaryocytic progenitors reinforces a phenotypic link between megakaryocytes and endothelial cells, which co-express numerous receptors, transcription factors and other signaling-associated factors [73]. The similarities become even more pronounced when considering hemogenic endothelium, which produces HSCs through EHT, due to the elevated expression of hematopoietic regulators which drive this process [74–76]. Megakaryocytes and endothelial cells are spatially close in hematopoietic vascular niches, their interactions conducted partially by CD31 [77]. Indeed, CD31’s absence impacts multiple aspects of megakaryopoiesis. It would therefore be worthwhile to determine whether CD31 deficiency impacts ancestral HPCs as well as their megakaryocytic progeny. Hematopoietic progenitor cell retention in the fetal liver vascular niche may consequently be severely impaired [68], potentially adding a new functional dimension to CD31 expression on CMPs and MEPs, as well as an immunophenotyping application.

One of the questions raised by our studies was whether the immunophenotypic CMP compartment actually contains single progenitor cells with the ability to produce granulocyte/monocyte, megakaryocyte and erythroid cells, thereby being defined as true Common Myeloid Progenitors. The alternative is that the CMP compartment solely comprises a heterogeneous population of monopotent or bipotent progenitors. Our single cell myeloid OP9 co-culture assays offer evidence that a small minority (<20%) of single isolated CMPs yield megakaryocytes, erythrocytes and granulocytes/monocytes, as would be expected for a true CMP. Therefore, we provide evidence that supports the existence of the CMP as a rare population within the fetal liver.

The CMP appears to be far scarcer than previously understood and it is likely that the LSK Multipotent Progenitor fractions may dominate in terms of common myeloid ancestry, particularly as they yield greater numbers of CFU-GEMMs than immunophenotypic CMPs. Indeed, we observed that fetal liver LSK HSCs and MPPs appeared to be the ancestors of the LK hematopoietic progenitors, at least *in vitro*. Contrastingly, we were unable to reproduce a similar myeloid progenitor hierarchy following culture of yolk sac cells. Nonetheless, several studies have suggested that at least some definitive hematopoietic stem and progenitor cells located in the embryo proper do not arise *de novo*, but instead originate from the yolk sac [75,78–80]. Therefore, it is possible that EMPs could be responsible to some extent for the establishment of a fetal liver hematopoietic progenitor hierarchy, including our populations of interest: the CD150^+^ MEPs and CMPs. Our results would suggest that this is only achieved after fetal liver colonization and differentiation in this supportive niche, yielding fetal liver hematopoietic stem and progenitor cells which, in turn, produce myeloid-restricted progenitors.

Our intent in this study was to delineate different fetal liver myeloid progenitor compartments, in order to provide a method to examine homeostatic developmental hematopoiesis and hematopoietic disease models. Indeed chromosomal translocations such as *AML1-ETO*, *PML-RARA* and *CBFβ-MYH11*, which cause childhood Acute Myeloid Leukemia (AML), frequently arise *in utero* as demonstrated by the high prevalence of such mutations in neonatal blood samples [34–36], and may therefore impact fetal hematopoiesis. Furthermore, Ye *et al* demonstrated that the initiation of AML requires partial myeloid differentiation by transformed CMPs to GMPs, highlighting how an understanding of the myeloid progenitor hierarchy facilitates examination of the origins and progression of malignant hematopoietic disorders [16]. To demonstrate the application of our fetal liver myeloid progenitor scheme, we analyzed the impact of deleting *Runx1* (a mutation which causes a megakaryocytic differentiation block comparable to FPD/AML [38,46,48]) on the specification and differentiation of these compartments. Akin to Behrens *et al* [81] in the adult, we observed deleting *Runx1* causes a block in fetal liver erythroid differentiation and pinpointed this block to CD150 downregulation in immunophenotypic MEPs (Fig 7B). We also observed decreased erythroid specification in the *Runx1-del/del* CD150^+^ CD31^low^ CMP fraction, and determined that this may be due to a failure to upregulate an erythroid transcriptional network in the transition from CD31^high^ to CD31^low^ CMP. We are therefore able to gather a substantial amount of phenotypic information and also gain mechanistic insights from transcriptome analyses.

We also evaluated the impact of removing the dominant RUNX1C isoform on fetal liver myelopoiesis. As we previously described in the adult, RUNX1C-null *P1-MRIPV/MRIPV* mice do not appear to have impaired megakaryocytic/erythroid differentiation [39]. Nonetheless, the fetal liver CD150^+^ CD31^-^ MEP fraction was expanded, partially recapitulating the *Runx1*-null erythroid lineage phenotype. However, *P1-MRIPV/MRIPV* fetal liver had reduced megakaryocytic specification, with the CD150^+^ CD31^high^ CMP-to-MkP transition being the most impaired pathway. We did not observe this in *Runx1*-null fetal liver; in fact megakaryocytic output increased in the absence of total RUNX1, as observed in adult hematopoiesis [39,81]. This difference may explain the apparently contradictory finding by Kuvardina *et al* [82] that RUNX1 promotes megakaryocytic specification; RUNX1C may have a non-redundant function in megakaryocytic specification, whereas RUNX1B is either necessary or sufficient for megakaryocytic and erythroid maturation. Our myeloid hematopoietic progenitor scheme has allowed us to identify the cells of interest which perpetuate this hematological imbalance. Such a technique could therefore be applied to understanding the cells of origin in familial thrombocytopenia, or a recently described case of *RUNX1*-deleted Congenital Amegakaryocytic Thrombocytopenia [83]. Moreover, our new protocol for prospective isolation of myeloid hematopoietic progenitors could be applied more broadly to other congenital or somatic genetic disorders which manifest *in utero*, as well as to analyzing lineage fate decisions in normal fetal liver hematopoiesis.

## Materials and methods

### Animal husbandry, timed matings and tissue collection

*Runx1-P1-GFP*::*P2-hCD4*, *Runx1-P1-MRIPV*, *Runx1-flox*::*Vav1-Cre*, *Gf1i-GFP* and *Gfi1b-GFP* mice have been described [17,38,39,84,85]. Dual-reporter *Runx1-P1-GFP*::*P2-RFP* chimeric mouse lines were generated by transfecting *P1-GFP* embryonic stem cells (ESCs) [17] with a *P2-RFP* targeting construct [86,87], screening for heterozygote knock-in lines targeting the same allele, and injecting correctly targeted ESCs into C57BL6J blastocysts.

*Runx1 P1-GFP*::*P2-hCD4*, *P1-GFP*::*P2-RFP*, *P1-MRIPV*, *Runx1 flox*::*Vav1-Cre*, *Gfi1-GFP* and *Gfi1b-GFP* mice were backcrossed with C57BL/6 mice for at least 10 generations and were housed in specific pathogen free cages with environmental enrichment. To trace *Runx1* promoter activity, timed matings were set up between *Runx1 P1-GFP*::*P2-hCD4* or *P1-GFP*::*P2-RFP* male mice and wild type (WT) ICR female mice. To produce *Runx1 P1-MRIPV/MRIPV* or *Runx1 flox/flox*::*Vav1-Cre* embryos (plus wild type and heterozygous control littermates), timed matings were set up between *Runx1 P1-MRIPV/+* mice or between *Runx1 flox/+*::*Vav1-Cre* males and *Runx1 flox/+* females. To trace *Gfi1* and *Gfi1b* expression, timed matings were set up between *Gfi1-GFP* or *Gfi1b-GFP* male mice and WT ICR female mice. Embryos were harvested on embryonic day (E) 12.5, 13.5, 14.5 or 16.5 and the fetal livers dissected for flow cytometric or histological analysis. For yolk sac experiments, embryos were harvested on E9.5 and the yolk sacs dissected for flow cytometric analysis, or for explant or pro-myeloid culture (as described below). Approximately 10^4^ cells were kept for genotyping by PCR (oligonucleotides listed in S1 Table).

All animal work was performed under regulations governed by UK Home Office Legislation under the Animals (Scientific Procedures) Act 1986 and was approved by the Animal Welfare and Ethics Review Body of the Cancer Research UK Manchester Institute.

### Fluorescence Activated Cell Sorting (FACS)

Details of FACS reagents and combinations used for each analysis are listed in S2 and S3 Tables. Prior to flow sorting or analysis of HPCs in E12.5, E13.5, E14.5 or E16.5 fetal liver, red blood cell lysis was performed as described [18]. Dead cells were excluded using 1μg/ml Hoechst 33258 (ThermoFisher Scientific); gates were positioned based on Full Minus One controls. Cells were analyzed using a LSR-II or LSR-II Fortessa analyzer (BD Biosciences) or a NovoCyte (ACEA Biosciences Inc.). Cells were sorted using a FACSAria-II, FACSAria-III or Influx cell sorter (BD Biosciences).

### Cell culture

#### OP9 co-culture

Mouse OP9 stromal cells were maintained in culture as described [18]. For bulk myeloid OP9 co-culture, fetal liver hematopoietic progenitors (2000 cells/ml) were seeded on OP9 cells (5000/ml). Myeloid OP9 co-culture medium has been described [18]. For single cell OP9 co-culture, single hematopoietic progenitor cells were directly sorted into 96-well plate wells containing 100μl myeloid OP9 co-culture medium and approximately 500 OP9 cells. Co-cultured cells were incubated in 5% CO_2_ and 5% O_2_ at 37°C for 7 days (5 days for single MEPs) before positive wells were assessed by microscopy and FACS analyzed for myeloid cell surface marker expression (TER119, CD41, GR1, CD11B, CKIT as detailed in S3 Table). For single cell cultures, the threshold for being counted as containing erythroid, megakaryocytic or granulocytic/monocytic cells was >2% TER119^+^, CD41^+^ or CD11b^+^ GR1^+^ cells respectively.

#### Hematopoietic colony-forming assays

Methylcellulose-based CFU-C assays were performed by plating 200 MEPs, 50 CMPs, 50 LSK HSPCs, 200 GMPs, 500 MkPs or 1000 cKit^-^ cells per dish in MethoCult GF M3534 (StemCell Technologies) supplemented with Penicillin-Streptomycin (100U/ml Penicillin, 100μg/ml Streptomycin, Sigma-Aldrich), 2U/ml erythropoietin (Eprex, Janssen-Cilag Ltd), medium conditioned by a cell line producing thrombopoietin (TPO) (1% final concentration), 5ng/ml IL11 (R&D Systems) and 10ng/ml Flt3 Ligand (Peprotech). For single cell CFU-C assays, single MEPs were directly sorted into 96-well plate wells containing 100μl MethoCult media as described above. CFUes were scored after 3–4 days and other colonies after 8 days under a microscope (DM IL, Leica).

#### Short-term culture of fetal liver hematopoietic stem and progenitor cells

Purified fetal liver MEPs, CMPs and LSK hematopoietic stem/progenitor cells were cultured at 37°C in 5% CO_2_ and atmospheric O_2_ for 14 hours (MEPS and CMPs) or 20 hours (LSK HSPCs) in pro-myeloid medium (IMDM supplemented with 10% Fetal Bovine Serum (FBS) for Mouse Myeloid Colony-Forming Cells (StemCell Technologies), 10% Protein-free hybridoma medium (PFHM-II, Gibco), 180μg/ml Transferrin (Roche Diagnostics), 0.45mM monothioglycerol (MTG, Sigma-Aldrich), 50ng/ml ascorbic acid (Sigma-Aldrich), 2mM L-glutamine (ThermoFisher Scientific), Penicillin-Streptomycin, 4U/ml erythropoietin, 5ng/ml IL11, 10ng/ml IL6 (R&D Systems), 10ng/ml M-CSF (R&D Systems) and medium conditioned by cell lines producing IL3, GMCSF, TPO and Stem Cell Factor (SCF) (1% final concentration). Cells were then harvested and FACS analyzed for hematopoietic stem/progenitor markers as described in S3 Table.

#### Short-term culture of yolk sac cells

Yolk sacs were dissected from E9.5 embryos and either cultured as explants as described [88] or dissociated with 1mg/ml Collagenase-Dispase/Phosphate Buffered Saline (PBS) (Roche) at 37°C for 20 minutes. Yolk sac cell suspensions were then either FACS analyzed for hematopoietic stem/progenitor markers as described in S3 Table (day 0) or cultured in pro-myeloid medium (see above) for 24 or 48 hours. After 48 hours, explant-cultured yolk sac cells were dissociated by trypsinization and either FACS analyzed for hematopoietic stem/progenitor markers (see S3 Table) immediately or following culture in pro-myeloid medium for a further 24 hours.

#### Megakaryocyte culture of fetal liver

Following red blood cell lysis, unfractionated fetal liver cells were cultured at 37°C in 5% CO_2_ and atmospheric O_2_ for 7 days at a density of 10^5^ cells per ml in pro-megakaryocyte medium (IMDM supplemented with 10% FBS for Mouse Myeloid Colony-Forming Cells, 2mM L-glutamine, Penicillin-Streptomycin, 0.45mM MTG, 20ng/ml IL6, 50ng/ml IL11 and medium conditioned by cell lines producing IL3 and TPO (1% final concentration). Cells were then harvested and FACS analyzed for CD41 and CD42d expression as described in S3 Table.

### Cytospin analysis

Up to 50,000 cultured fetal liver cells were suspended in 150μl PBS and immobilized on twin frosted glass microscope slides (Fisher Scientific) by cytospin at 200rpm, low acceleration for 5 minutes in a Shandon Cytospin3 (Thermo Scientific). Air-dried slides were submerged in May-Grünwald Eosin methylene blue Q Path stain (VWR) for 3 minutes, rinsed in tap water and submerged in 5% Giemsa’s stain (VWR) for 20 minutes. Slides were subsequently air dried, mounted and scanned using the Pannoramic 250 Flash III (3DHISTECH). Images were acquired with the Pannoramic 250 software and analyzed with the Pannoramic Viewer software (3DHISTECH).

### Immunofluorescent staining and imaging of fetal liver sections

Dissected fetal livers were fixed in 4% Paraformaldehyde (PFA) overnight, before they were soaked in 30% sucrose and mounted in OCT compound. 10μm sections were prepared using a cryostat. The sections were incubated in blocking buffer (PBS with 10% FBS, 0.05% Tween20 and 10% goat serum (DAKO)) for 1 hour before the sections were incubated with primary antibodies at 4°C overnight in blocking buffer.

Primary antibodies used in this study were rabbit anti-GFP (598, polyclonal, MBL) (1/200); and purified rat anti-mouse CD31 (553370, MEC13.3, BD Biosciences) (1/100).

Sections were washed three times in PBST (PBS with 0.05% Tween20) for 15 minutes each and then incubated with fluorochrome-conjugated secondary antibody at room temperature for 1 hour.

Secondary antibodies used in this study include Alexa Fluor 488 Goat Anti-Rat IgG (A11006, Life Technologies); and Alexa Fluor 647 F(ab')_2_ Fragment of Goat Anti-Rabbit IgG (H+L) (A21246, Life Technologies). All secondary antibodies were used at 1/400 dilution.

Sections were further washed three times in PBS and mounted using Prolong Gold anti-fade medium with DAPI (Life Technologies). Images (of Alexa Fluor 488, Alexa Fluor 647, DAPI and endogenous RFP) were taken using a low-light time lapse microscope (Leica) using the Metamorph imaging software and processed using ImageJ.

### Western blot analysis

Whole cell protein extracts, purified using RIPA lysis buffer, were quantitated by the Bradford assay (Protein Assay Dye Reagent, Bio-Rad) on the Glomax Multi Detection System (Promega) and loaded alongside the SeeBlue Plus2 Prestained Standard (ThermoFisher Scientific) for electrophoretic separation on NuPAGE 4–12% Bis-Tris gels using the Novex Mini Cell system (ThermoFisher Scientific). Protein transfer was performed to nitrocellulose membranes using the iBlot system (ThermoFisher Scientific) and membranes were probed with anti-RUNX (EPR3099, Abcam) and anti-beta-actin (AC-15, Sigma-Aldrich) primary antibodies, followed by Horseradish Peroxidase (HRP)-conjugated goat anti-rabbit and goat anti-mouse (ThermoFisher Scientific) secondary antibodies respectively, in the iBind Western System (ThermoFisher Scientific). HRP activity was detected using Amersham ECL Prime Western Blotting Detection Reagent, imaged using the BioRad ChemiDoc Touch Imaging System and analyzed with BioRad Image Lab Software Version 6.

### Gene expression by quantitative PCR

RNA was extracted using the RNeasy Plus Micro Kit (QIAGEN) and complementary DNA was synthesized using the High-Capacity cDNA Reverse Transcription Kit (ThermoFisher Scientific). Quantitative PCR (qPCR) was performed using Universal ProbeLibrary assays (Roche); primers and probes are listed in S4 Table. Expression values were normalized to *beta-actin* (*Actb*).

### Single cell RNA sequencing

E14.5 *P1-GFP*::*P2-hCD4/+* fetal livers were prepared for flow sorting as described above. Single *P2-hCD4*^*-/+*^ MEPs and CMPs were sorted into 384-well plates containing lysis buffer and snap frozen. Libraries were prepared using a modified version of the Smart-Seq2 protocol [89]. Briefly, cDNA was prepared using a Mantis platform (Formulatrix) and quantified with quantIT picogreen reagent (Thermo Fisher). Dual indexed sequencing libraries were prepared from 0.1ng cDNA using an Echo525 automation system (Labcyte) in miniaturized reaction volumes. The library pool was quantified by qPCR using a Library Quantification Kit for Illumina sequencing platforms (Kapa Biosystems). Paired-end 75bp sequencing was carried out by clustering 1.5pM of the library pool on a NextSeq 500 sequencer (Illumina).

Base call files generated from the NextSeq 500 sequencing run were converted to the FASTQ format with the bcl2fast converter (Illumina). All FASTQ files corresponding to the same sample (derived from separately sequenced lanes) were then merged into a single FASTQ file (one per sample). Read trimming was performed with trimmomatic (v0.36) with the following settings “CROP:75 HEADCROP:5 SLIDINGWINDOW:20:20 MINLEN:36”. The mouse reference genome GRCm38 (version M12, Ensembl release 87) and the ERCC reference sequence (Thermo Fisher) were combined and used as the reference genome for sequencing alignment, performed using STAR (version 2.4.2a) [90]. The expression levels of 49,585 features annotated in the GENCODE mouse genome and 92 ERCC features were determined using HTSeq (version 0.6.1p1) [91]. The following parameters were specified for the HTSeq quantification: ‘—format = bam–stranded = no–type = exon’.

Single cell count data were loaded into the R environment (R version 3.4.0) as a SCESet object using the Scater package (version 1.4.0) [92]. Normalized gene expression values were taken from the default normalization performed by scater. Cells with fewer than 250,000 sequencing reads, more than 25% unmapped reads, and more than 15% ERCC content were removed. Low abundance genes (mean count <1) were excluded, as were overrepresented genes (>20% of total sequencing reads). Differentially expressed genes were identified using DESeq2 (version 1.16.1, Bioconductor). Prior to differential expression analysis, the data were filtered to remove genes with a dropout rate of higher than 75%; differential expression analysis was then performed using the function “DESeqDataSetFromMatrix” and by specifying the contrast of interest. The full scripts for the analysis of these data are available at https://github.com/m-zaki/CRUKMI_github/tree/master/JuliaDraper_PLOSgenetics.

The data discussed in this publication have been deposited in the NCBI Gene Expression Omnibus [93,94] and are accessible through GEO series accession number GSE107653.

### Statistical analysis

For FACS purified populations (MEPs, CMPs) a sample size of n = 1 refers to tissues pooled from embryos from one litter. For total fetal liver analyses (Western blot, total FL culture), a sample size of n = 1 refers to one embryo.

Data were evaluated using an Ordinary 2-way ANOVA and expressed as mean ± standard error of the mean (SEM). *P*<0.05 was considered statistically significant.

*P<0.05, **P<0.01, ***P<0.001, ****P<0.0001

## Supporting information

S1 Fig Lineage output of Lin^-^ Sca1^high^ cKit^high^ (LSK), GMP and MkP Hematopoietic Stem and Progenitor Cells (Relating to Fig 1). A. Differential CFU-C activity of E14.5 fetal liver cKit^-^ cells, GMPs and MkPs. N = 3.
B-F. Flow cytometric analysis of wild type (WT) E14.5 fetal liver GMPs and MkPs. B. Representative FACS plot of CD55/CD150 expression in GMPs. C. Quantitation of CD55^low^ CD150^low^ and CD55^mid/high^ GMPs. D-E. Representative FACS plots of total immunophenotypic MkP fraction (D) and CD55/CD150 expression in MkPs (E). F. Quantitation of CD55^-^ CD150^-^, CD55^+^ CD150^-^ and CD55^+^ CD150^+^ MkPs. N = 3.
G-H. Extended characterization of OP9 co-cultured CD55^-^ CD150^-^ MEPs. G. Representative FACS plots of CD11b/GR1 and C-KIT expression of day 7 cultures. H. Quantitation of CD11b^+^ GR1^+^ GM and C-KIT^+^ populations. N = 3.
I. Representative FACS plots of E14.5 fetal liver LSK populations.
J. Differential CFU-C activity of E14.5 fetal liver LSK CD150^+^ CD48^-^ Hematopoietic Stem Cells (HSCs), LSK CD150^-^ CD48^-^ MPPs (CD48^-^ MPPs), LSK CD48^+^ CD150^+^ MPPs and LSK CD48^+^ CD150^-^ MPPs. N = 3.
K-L. Short-term (20 hours) differentiation of wild type LSK hematopoietic stem and progenitor cells in pro-myeloid liquid culture. K. Representative FACS plots of cultured LSK hematopoietic stem and progenitor cells. L. Proportions of immunophenotypic LK and LSK hematopoietic stem and progenitor cells in short-term cultures. Top: total Lin and cKit populations; Middle: Differential CD150/CD48-expressing LSK populations; Bottom: CMP, MEP, GMP and MkP populations. N = 4.
M-N. Short-term differentiation of wild type E9.5 yolk sac cells, either directly in pro-myeloid liquid culture or explant culture followed by pro-myeloid liquid culture. M. Representative FACS plots of uncultured and cultured E9.5 yolk sac cells. N. Proportions of immunophenotypic LK and LSK populations from yolk sac cells, with or without culture. Top: total Lin and cKit populations; Bottom: immunophenotypic CMP, MEP, GMP, MkP and LK CD16/32- CD150- CD41+ populations. E9.5 YS, N = 9; 24hr Pro-myeloid, N = 5; 48hr Pro-myeloid, N = 4; Explant, N = 8.
(PDF)

S2 Fig Lineage output of E12.5 and E13.5 Fetal Liver *Runx1-P1-GFP*::*P2-hCD4* MEPs (Relating to Fig 2). A-B. Differential CFU-C activity of E12.5 (A, N = 3) and E13.5 (B, N = 4) fetal liver wild type, *P2-hCD4*^*-*^ and *P2-hCD4*^*+*^ MEPs. C-F. Lineage output of day 7 OP9 co-cultured fetal liver wild type, *P2-hCD4*^*-*^ and *P2-hCD4*^*+*^ MEPs.
C-D. Representative FACS plots of TER119 and CD41 expression in E12.5 (C) and E13.5 (D) cultured MEPs. E-F. Proportion of TER119^+^ erythroid and CD41^+^ megakaryocyte cells in E12.5 (E, N = 3) and E13.5 (F, N = 4) MEP cultures.
(PDF)

S3 Fig Lineage output of E12.5 and E13.5 Fetal Liver Runx1 *P1-GFP*::*P2-hCD4* CMPs (Relating to Fig 3). A-B. Differential CFU-C activity of E12.5 (A, N = 3) and E13.5 (B, N = 4) fetal liver wild type, *P2-hCD4*^*-*^ and *P2-hCD4*^*+*^ CMPs.
C-F. Lineage output of day 7 OP9 co-cultured fetal liver wild type, *P2-hCD4*^*-*^ and *P2-hCD4*^*+*^ CMPs. C-D. Representative FACS plots of CD11b/GR1 and TER119/CD41 expression in E12.5 (C) and E13.5 (D) cultured CMPs. E-F. Proportion of CD11b^+^ GR1^+^ granulocyte/monocyte, TER119^+^ erythroid and CD41^+^ megakaryocyte cells in E12.5 (E, N = 3) and E13.5 (F, N = 4) CMP cultures.
(PDF)

S4 Fig Characterization of CD31^-/+^ MEPs and CD31^low/high^ CMPs (Relating to Fig 4). A. Principal component analysis of the single cell RNA sequencing expression data from CD41^-^ CD150^+^ *P2-hCD4*^*-*^ and *P2-hCD4*^*+*^ MEPs and CMPs (P2- MEP, P2+ MEP, P2- CMP, P2+ CMP). Top: cells are color-coded according to the sorted population. Middle and Bottom plots: cells are color-coded according to their expression of *Klf1* (2^nd^ row), *Fli1* (3^rd^ row) and *Spi1* (bottom row).
B. Violin plots of expression of selected cell surface marker genes in single P2- and P2+ MEPs, determined by RNA sequencing.
C. Representative FACS plots characterizing CD31, CD45, CD48, Endoglin, CD11b, Tie2, Flk1, CD44, CD144, CD24, CD29, CD43, CD140a and CD184 expression in *Runx1 P2-hCD4*^*-*^ and *P2-hCD4*^*+*^ E14.5 fetal liver cells.
D. Numbers of DAPI^+^, CD31^+^, *P1-GFP*^*+*^ and *P2-RFP*^*+*^ cells in E14.5 *P1-GFP*::*P2-RFP* FL sections. N = 3 independent samples.
E. CD48/CD45 expression in wild type E14.5 CD41^-^ CD150^+^ MEPs and CMPs.
F. CD31/*GFI1b-GFP* expression in *Gfi1b-GFP/+* E14.5 CD41^-^ CD150^+^ MEPs and CMPs.
G. CD31/*GFI1-GFP* expression in *Gfi1-GFP/+* E14.5 CD41^-^ CD150^+^ MEPs and CMPs.
H-I. Lineage output of single cultured wild type CD31^-^ and CD31^+^ MEPs in MethoCult semi-solid myeloid culture medium (H) and OP9 co-cultures (I).
J. Lineage output of single cultured wild type CD31^low^ and CD31^high^ CMPs in OP9 co-cultures.
K-N. Short-term (14 hours) differentiation of wild type CD31^-/+^ MEPs and CD31^low/high^ CMPs in pro-myeloid liquid culture. K-L. Representative FACS plots of cultured MEPs (K) and CMPs (L). M. Proportions of immunophenotypic MkPs and MEPs in short-term MEP cultures, N = 6. N. Proportions of immunophenotypic MkPs, MEPs, GMPs and CMPs in short-term CMP cultures, N = 4.
(PDF)

S5 Fig Characterization of E16.5 Fetal Liver CMPs and MEPs (Relating to Fig 4). A-B. Expression of *Runx1-P1-GFP* and *P2-hCD4* in *P1-GFP*::*P2-hCD4/+* E16.5 fetal liver CD41^-^ CD150^+^ MEPs and CMPs. A. Representative FACS plots. B. Quantitation of the proportions of *P1*^*-*^ *P2*^*-*^, *P1*^*+*^ *P2*^*-*^, *P1*^*+*^ *P2*^*+*^ and *P1*^*-*^ *P2*^*+*^ MEPs and CMPs as a percentage of total live red blood cell lysed E16.5 fetal liver cells. N = 3.
C-D. Differential CFU-C activity of *P2-hCD4*^*-*^ and *P2-hCD4*^*+*^ E16.5 fetal liver CD41^-^ CD150^+^ MEPs (C) and CMPs (D). N = 5.
E-H. Lineage output of day 7 OP9 co-cultured *P2-hCD4*^*-*^ and *P2-hCD4*^*+*^ CD41^-^ CD150^+^ MEPs and CMPs. E-F. Representative FACS plots of TER119/CD41 and CD11b/GR1. G-H. Proportions of granulocyte/monocyte, erythroid and megakaryocyte cells. N = 5.
I. Representative FACS plots of CD31 expression in *P1-GFP*::*P2-hCD4/+* E16.5 FL *P2-hCD4*^*-*^ and *P2-hCD4*^*+*^ LK cells.
J. Representative FACS plots of CD31/CD45 expression in wild type E16.5 fetal liver CD41^-^ CD150^+^ MEPs and CMPs.
K-L. Differential CFU-C activity in wild type E16.5 fetal liver CD31^-/+^ MEPs (K) and CD31^low/high^ CMPs (L). N = 5.
M-P. Lineage output of OP9 co-cultured MEPs and CMPs. M. Representative FACS plots of TER119 and CD41 expression of day 7 OP9 co-cultured wild type E16.5 MEPs. N. Representative FACS plots of CD11b, GR1, TER119 and CD41 expression of day 7 OP9 co-cultured wild type E16.5 CMPs. O. Proportion of TER119^+^ erythroid cells and CD41^+^ megakaryocyte cells in day 7 OP9 wild type E16.5 MEP co-cultures. P. Proportions of granulocytes/monocytes, erythroid cells and megakaryocytes in day 7 OP9 wild type E16.5 CMPs co-cultures. N = 5.
(PDF)

S6 Fig Impaired Mk/Ery Maturation in *Runx1-*null Fetal Liver (Relating to Fig 5). A. RUNX1/ACTINB Western blot of total protein extract from wild type, *Runx1-del/+* and *Runx1-del/del* E14.5 fetal liver. Representative of 3 independent experiments.
B-E. Characterization of erythroid lineage subsets S0-S5 in wild type, *Runx1-del/+* and *Runx1-del/del* E14.5 fetal liver. B. Representative FACS plots of CD71/TER119 expression. C-E. Quantitation of S0 (CD71^-/low^ TER119^-^), S1 (CD71^high^ TER119^-^), S2 (CD71^high^ TER119^low^) (C), S3 (CD71^high^ TER119^high^) (D), S4 (CD71^low^ TER119^high^) and S5 (CD71^-^ TER119^high^) (E) erythroid populations. WT N = 10, *Runx1-del/+* N = 9, *Runx1-del/del* N = 3.
F-G. Characterization of CD41^high^ CD42d^-^ and CD41^high^ CD42d^+^ megakaryocytes from day 7 total fetal liver cultures (wild type, *Runx1-del/+* and *Runx1-del/del*). F. Representative FACS plots of CD41/CD42d expression. G. Quantitation of megakaryocytic CD41^high^ CD42d- and mature megakaryocyte CD41^high^ CD42d^+^ fractions. WT N = 10, *Runx1-del/+* N = 9, *Runx1-del/del* N = 3.
H. Morphologic analysis of day 7 cultured megakaryocytes, stained with May-Grünwald Giemsa reagent.
(PDF)

S7 Fig Characterization of *Runx1-*null progenitors (Relating to Fig 5). A-B. Characterization of myeloid progenitor populations in wild type, *Runx1-del/+* and *Runx1-del/del* E14.5 FL. A. Representative FACS plots of MEP, CMP, GMP and MkP populations. B. Quantitation of myeloid hematopoietic progenitors. N = 6.
C-F. Differential CFU-C activity of CD31^-^ MEPs (C), CD31^+^ MEPs (D), CD31^low^ CMPs (E) and CD31^high^ CMPs (F). N = 5.
G-I. Lineage output of bulk OP9 co-cultured wild type, *Runx1-del/+* and *Runx1-del/del* E14.5 fetal liver CD31^-^ and CD31^+^ MEPs. G. Representative FACS plots of TER119/CD41 expression. H-I. Quantitation of TER119^+^ erythroid and CD41^+^ megakaryocyte cells in CD31^-^ MEP (H) and CD31^+^ MEP (I) co-cultures. N = 4.
J-K. Lineage output of bulk OP9 co-cultured wild type, *Runx1-del/+* and *Runx1-del/del* E14.5 fetal liver CD31^high^ CMPs. J. Representative FACS plots of CD11b/GR1 and TER119/CD41 expression. K. Quantitation of CD11b^+^ GR1^+^ granulocyte/monocyte, TER119^+^ erythroid and CD41^+^ megakaryocyte cells in CD31^high^ CMP co-cultures. N = 4.
L-P. Lineage output of single OP9 co-cultured wild type, *Runx1-del/+* and *Runx1-del/del* E14.5 fetal liver CMPs. L. Quantitation of CD11b^+^ GR1^+^ granulocyte/monocyte, TER119^+^ erythroid and CD41^+^ megakaryocyte populations in CD31^low^ CMPs. M. Ternary plots displaying proportions of granulocyte/monocyte, erythroid and megakaryocyte cells in each positive well for CD31^high^ CMPs. N. Quantitation of CD11b^+^ GR1^+^ granulocyte/monocyte, TER119^+^ erythroid and CD41^+^ megakaryocyte populations in CD31^high^ CMP cultures. O. Proportions of unilineage (Ery, Mk and GM only) and multilineage (Mk + Ery, Mk + GM, Ery + GM and GEMM) wells derived from CD31^high^ CMPs. P. TER119 Median Fluorescence Intensity of cells of TER119^+^ cells. N = 3.
Q-R. Quantitative PCR analysis of the expression of selected RUNX1-target and/or hematopoietic lineage-associated genes in CD31^low^ and CD31^high^ CMPs. Q. Expression of RUNX1-associated transcription factors. R. Expression of other megakaryocytic and erythroid markers. N = 3
(PDF)

S8 Fig Mk/Ery maturation in *Runx1-P1-MRIPV* Fetal Liver (Relating to Fig 6). A. RUNX1/ACTINB Western blot of total protein extract from wild type, *Runx1-P1-MRIPV/+* and *Runx1-P1-MRIPV/MRIPV* E14.5 fetal liver. Representative of 3 independent experiments.
B-E. Characterization of erythroid lineage subsets S0-S5 in wild type, *Runx1-P1-MRIPV/+* and *Runx1-MRIPV/MRIPV* E14.5 fetal liver. B. Representative FACS plots of CD71/TER119 expression. C-E. Quantitation of S0, S1 and S2 (C), S3 (D), and S4 and S5 (E) erythroid populations. WT N = 12, *Runx1-P1-MRIPV/+* N = 9, *Runx1-P1-MRIPV/MRIPV* N = 9.
F-G. Characterization of CD41^high^ CD42d^-^ and CD41^high^ CD42d^+^ megakaryocytes from day 7 total fetal liver cultures (*Runx1* wild type, *P1-MRIPV/+* and *P1-MRIPV/MRIPV*). F. Representative FACS plots of CD41/CD42d expression. G. Quantitation of megakaryocyte CD41^high^ CD42d- and mature megakaryocyte CD41^high^ CD42d^+^ fractions. WT N = 12, *Runx1-P1-MRIPV/+* N = 9, *Runx1-P1-MRIPV/MRIPV* N = 9.
(PDF)

S9 Fig Characterization of *Runx1-P1-MRIPV* Fetal Liver progenitors (Relating to Fig 6). A-B. Characterization of myeloid progenitor populations in wild type, *Runx1-P1-MRIPV/+* and *Runx1-P1-MRIPV/MRIPV* E14.5 fetal liver. A. Representative FACS plots of MEP, CMP, GMP and MkP populations. B. Quantitation of myeloid hematopoietic progenitors. N = 7.
C-F. Differential CFU-C activity of CD31^-^ MEPs (C), CD31^+^ MEPs (D), CD31^low^ CMPs (E) and CD31^high^ CMPs (F). N = 5.
G-I. Lineage output of bulk OP9 co-cultured wild type, *Runx1-P1-MRIPV/+* and *Runx1-P1-MRIPV/MRIPV* E14.5 fetal liver CD31^-^ and CD31^+^ MEPs. G. Representative FACS plots of TER119/CD41 expression. H-I. Quantitation of TER119^+^ erythroid and CD41^+^ megakaryocyte cells in CD31^-^ MEP (H) and CD31^+^ MEP (I) co-cultures. N = 5.
J-M. Short-term (14 hours) differentiation of E14.5 wild type, *Runx1-P1-MRIPV/+* and *Runx1-P1-MRIPV/MRIPV* CD31^-/+^ MEPs and CD31^low/high^ CMPs in pro-myeloid liquid culture. J-K. Representative FACS plots of cultured CD31^low^ CMPs (J) and CD31^high^ CMPs (K). L-M. Proportions of immunophenotypic MkPs (L) and GMPs (M) in hematopoietic progenitor cultures. N = 3.
(PDF)

S1 Table Primers used for mouse genotyping PCRs. (DOCX)

S2 Table Details of flow cytometry reagents. (DOCX)

S3 Table Antibody combinations used for flow cytometric analysis and sorting (see S2 Table for clone and supplier details). (DOCX)

S4 Table Primers and probes used for qPCR. (DOCX)

S5 Table Differentially expressed genes in P2+ MEPs with respect to P2- MEPs. (XLSX)

S6 Table Differentially expressed genes in P2+ CMPs with respect to P2- CMPs. (XLSX)
