# Supplementary figures and images for: A novel prospective isolation of murine fetal liver progenitors to study in utero hematopoietic defects
Source: PLoS Genet. 2018 Jan 4;14(1):e1007127. doi: 10.1371/journal.pgen.1007127 (PMC5754050; doi:10.1371/journal.pgen.1007127)

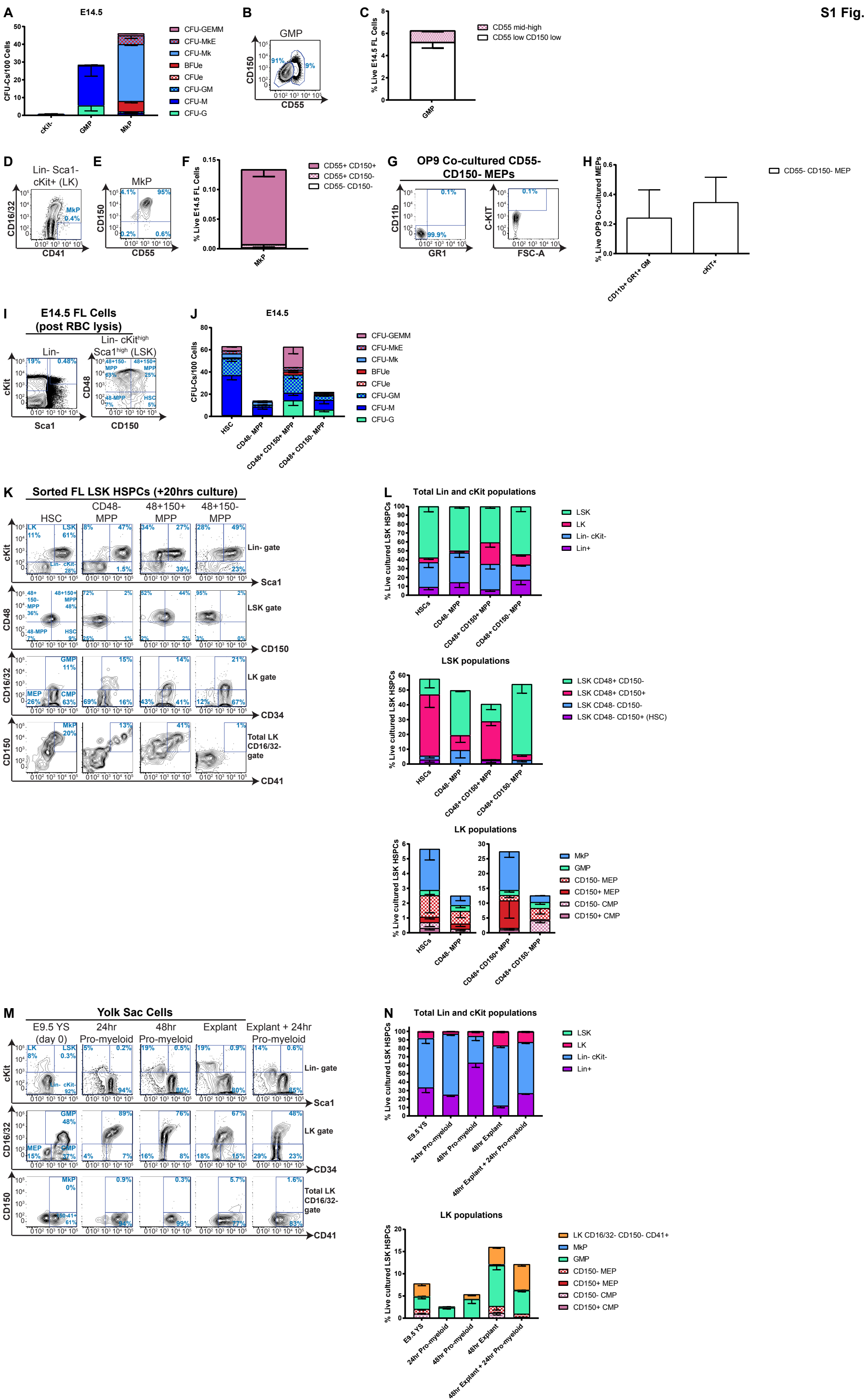

Supplement: S1 Fig — A. Differential CFU-C activity of E14.5 fetal liver cKit- cells, GMPs and MkPs. N = 3. B-F. Flow cytometric analysis of wild type (WT) E14.5 fetal liver GMPs and MkPs. B. Representative FACS plot of CD55/CD150 expression in GMPs. C. Quantitation of CD55low CD150low and CD55mid/high GMPs. D-E. Representative FACS plots of total immunophenotypic MkP fraction (D) and CD55/CD150 expression in MkPs (E). F. Quantitation of CD55- CD150-, CD55+ CD150- and CD55+ CD150+ MkPs. N = 3. G-H. Extended characterization of OP9 co-cultured CD55- CD150- MEPs. G. Representative FACS plots of CD11b/GR1 and C-KIT expression of day 7 cultures. H. Quantitation of CD11b+ GR1+ GM and C-KIT+ populations. N = 3. I. Representative FACS plots of E14.5 fetal liver LSK populations. J. Differential CFU-C activity of E14.5 fetal liver LSK CD150+ CD48- Hematopoietic Stem Cells (HSCs), LSK CD150- CD48- MPPs (CD48- MPPs), LSK CD48+ CD150+ MPPs and LSK CD48+ CD150- MPPs. N = 3. K-L. Short-term (20 hours) differentiation of wild type LSK hematopoietic stem and progenitor cells in pro-myeloid liquid culture. K. Representative FACS plots of cultured LSK hematopoietic stem and progenitor cells. L. Proportions of immunophenotypic LK and LSK hematopoietic stem and progenitor cells in short-term cultures. Top: total Lin and cKit populations; Middle: Differential CD150/CD48-expressing LSK populations; Bottom: CMP, MEP, GMP and MkP populations. N = 4. M-N. Short-term differentiation of wild type E9.5 yolk sac cells, either directly in pro-myeloid liquid culture or explant culture followed by pro-myeloid liquid culture. M. Representative FACS plots of uncultured and cultured E9.5 yolk sac cells. N. Proportions of immunophenotypic LK and LSK populations from yolk sac cells, with or without culture. Top: total Lin and cKit populations; Bottom: immunophenotypic CMP, MEP, GMP, MkP and LK CD16/32- CD150- CD41+ populations. E9.5 YS, N = 9; 24hr Pro-myeloid, N = 5; 48hr Pro-myeloid, N = 4; Explant, N = 8. (PDF) [file pgen.1007127.s001.pdf]

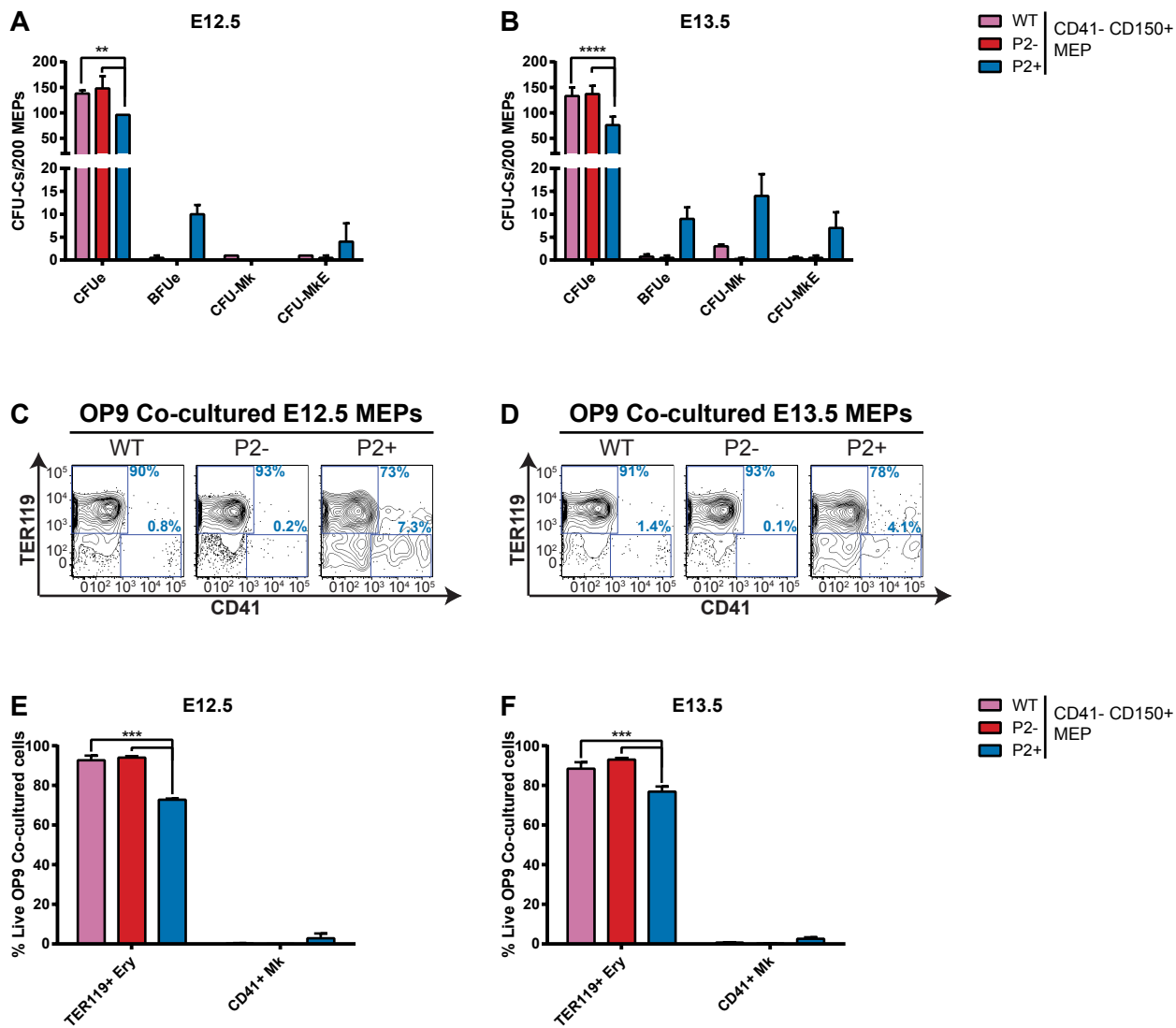

Supplement: S2 Fig — A-B. Differential CFU-C activity of E12.5 (A, N = 3) and E13.5 (B, N = 4) fetal liver wild type, P2-hCD4- and P2-hCD4+ MEPs. C-F. Lineage output of day 7 OP9 co-cultured fetal liver wild type, P2-hCD4- and P2-hCD4+ MEPs. C-D. Representative FACS plots of TER119 and CD41 expression in E12.5 (C) and E13.5 (D) cultured MEPs. E-F. Proportion of TER119+ erythroid and CD41+ megakaryocyte cells in E12.5 (E, N = 3) and E13.5 (F, N = 4) MEP cultures. (PDF) [file pgen.1007127.s002.pdf]

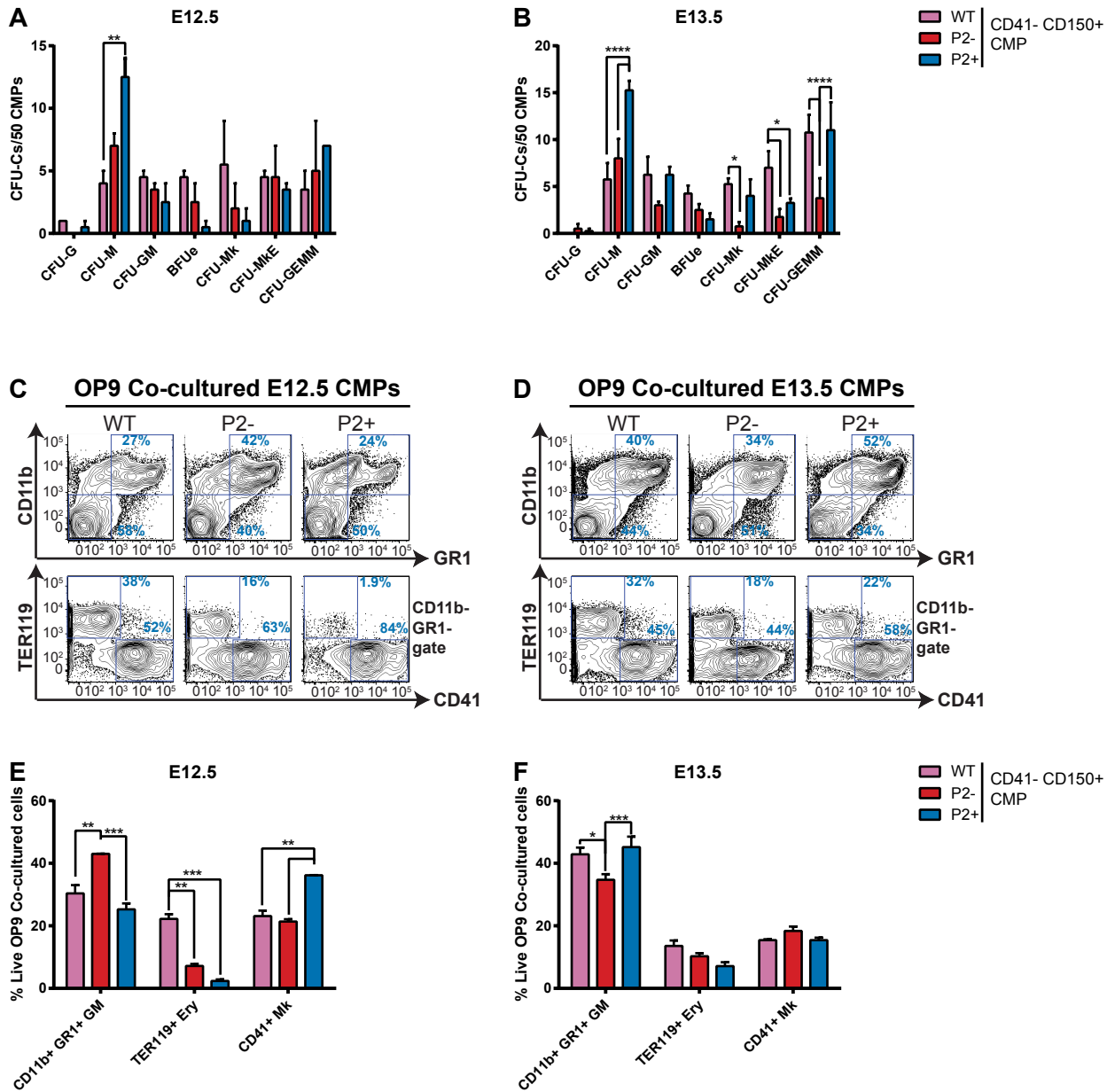

Supplement: S3 Fig — A-B. Differential CFU-C activity of E12.5 (A, N = 3) and E13.5 (B, N = 4) fetal liver wild type, P2-hCD4- and P2-hCD4+ CMPs. C-F. Lineage output of day 7 OP9 co-cultured fetal liver wild type, P2-hCD4- and P2-hCD4+ CMPs. C-D. Representative FACS plots of CD11b/GR1 and TER119/CD41 expression in E12.5 (C) and E13.5 (D) cultured CMPs. E-F. Proportion of CD11b+ GR1+ granulocyte/monocyte, TER119+ erythroid and CD41+ megakaryocyte cells in E12.5 (E, N = 3) and E13.5 (F, N = 4) CMP cultures. (PDF) [file pgen.1007127.s003.pdf]

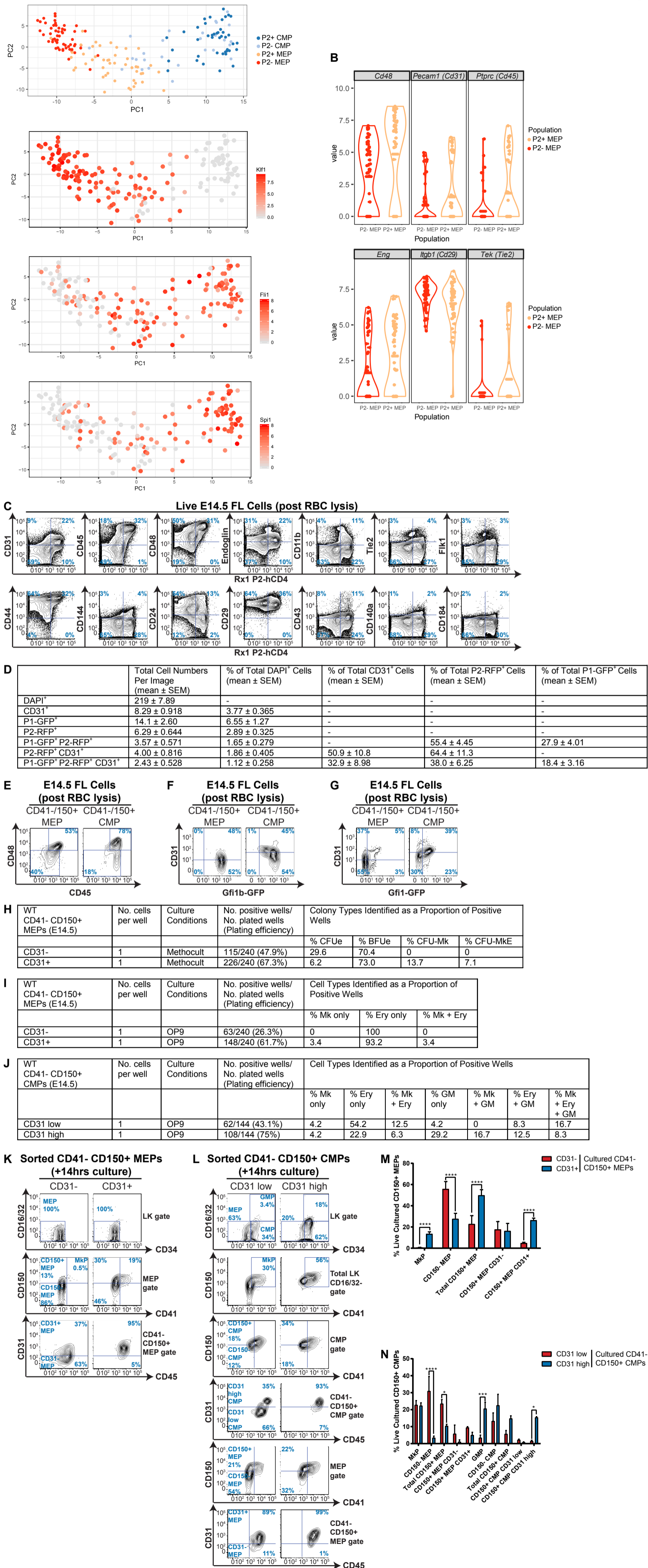

Supplement: S4 Fig — A. Principal component analysis of the single cell RNA sequencing expression data from CD41- CD150+ P2-hCD4- and P2-hCD4+ MEPs and CMPs (P2- MEP, P2+ MEP, P2- CMP, P2+ CMP). Top: cells are color-coded according to the sorted population. Middle and Bottom plots: cells are color-coded according to their expression of Klf1 (2nd row), Fli1 (3rd row) and Spi1 (bottom row). B. Violin plots of expression of selected cell surface marker genes in single P2- and P2+ MEPs, determined by RNA sequencing. C. Representative FACS plots characterizing CD31, CD45, CD48, Endoglin, CD11b, Tie2, Flk1, CD44, CD144, CD24, CD29, CD43, CD140a and CD184 expression in Runx1 P2-hCD4- and P2-hCD4+ E14.5 fetal liver cells. D. Numbers of DAPI+, CD31+, P1-GFP+ and P2-RFP+ cells in E14.5 P1-GFP::P2-RFP FL sections. N = 3 independent samples. E. CD48/CD45 expression in wild type E14.5 CD41- CD150+ MEPs and CMPs. F. CD31/GFI1b-GFP expression in Gfi1b-GFP/+ E14.5 CD41- CD150+ MEPs and CMPs. G. CD31/GFI1-GFP expression in Gfi1-GFP/+ E14.5 CD41- CD150+ MEPs and CMPs. H-I. Lineage output of single cultured wild type CD31- and CD31+ MEPs in MethoCult semi-solid myeloid culture medium (H) and OP9 co-cultures (I). J. Lineage output of single cultured wild type CD31low and CD31high CMPs in OP9 co-cultures. K-N. Short-term (14 hours) differentiation of wild type CD31-/+ MEPs and CD31low/high CMPs in pro-myeloid liquid culture. K-L. Representative FACS plots of cultured MEPs (K) and CMPs (L). M. Proportions of immunophenotypic MkPs and MEPs in short-term MEP cultures, N = 6. N. Proportions of immunophenotypic MkPs, MEPs, GMPs and CMPs in short-term CMP cultures, N = 4. (PDF) [file pgen.1007127.s004.pdf]

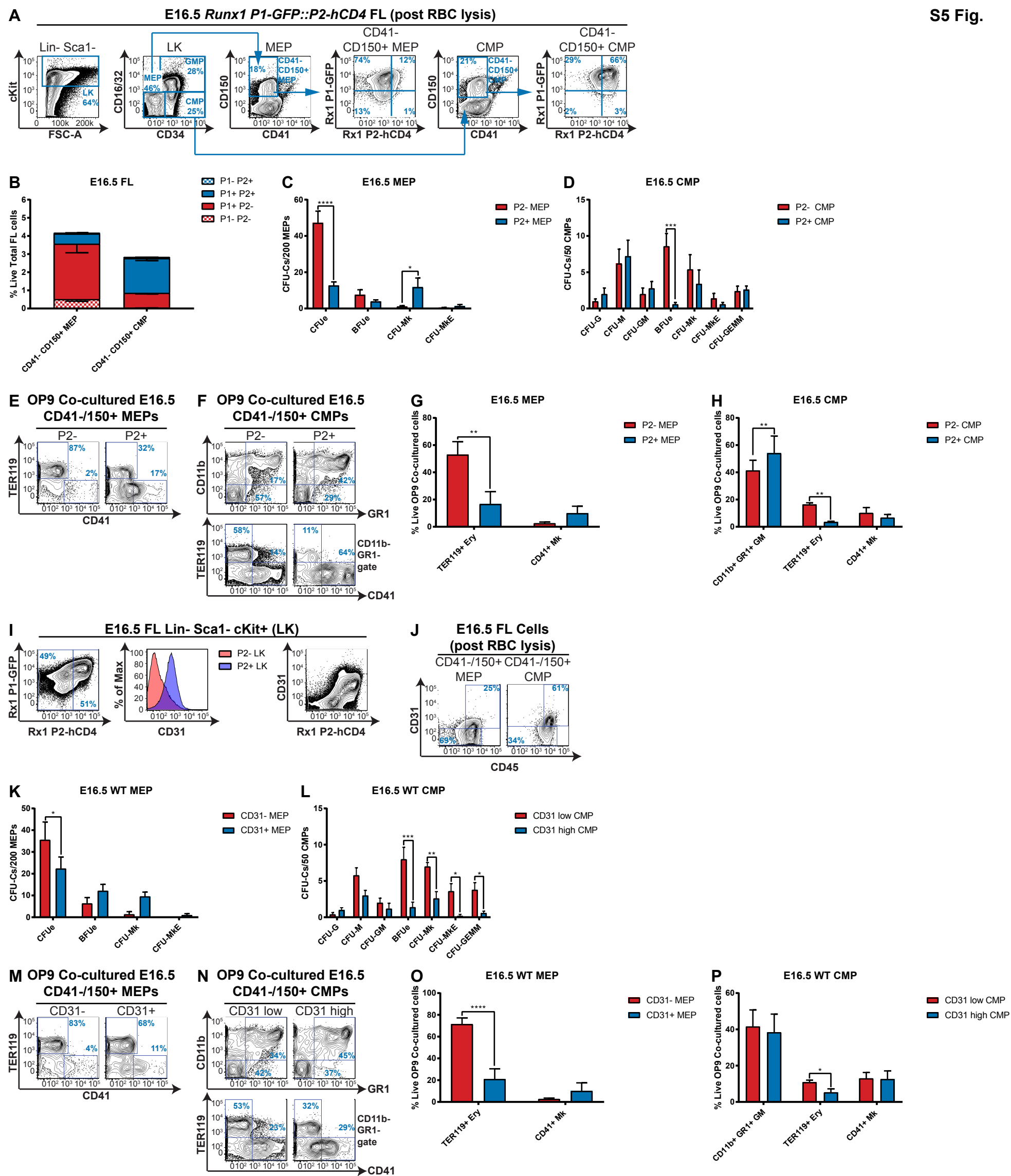

Supplement: S5 Fig — A-B. Expression of Runx1-P1-GFP and P2-hCD4 in P1-GFP::P2-hCD4/+ E16.5 fetal liver CD41- CD150+ MEPs and CMPs. A. Representative FACS plots. B. Quantitation of the proportions of P1- P2-, P1+ P2-, P1+ P2+ and P1- P2+ MEPs and CMPs as a percentage of total live red blood cell lysed E16.5 fetal liver cells. N = 3. C-D. Differential CFU-C activity of P2-hCD4- and P2-hCD4+ E16.5 fetal liver CD41- CD150+ MEPs (C) and CMPs (D). N = 5. E-H. Lineage output of day 7 OP9 co-cultured P2-hCD4- and P2-hCD4+ CD41- CD150+ MEPs and CMPs. E-F. Representative FACS plots of TER119/CD41 and CD11b/GR1. G-H. Proportions of granulocyte/monocyte, erythroid and megakaryocyte cells. N = 5. I. Representative FACS plots of CD31 expression in P1-GFP::P2-hCD4/+ E16.5 FL P2-hCD4- and P2-hCD4+ LK cells. J. Representative FACS plots of CD31/CD45 expression in wild type E16.5 fetal liver CD41- CD150+ MEPs and CMPs. K-L. Differential CFU-C activity in wild type E16.5 fetal liver CD31-/+ MEPs (K) and CD31low/high CMPs (L). N = 5. M-P. Lineage output of OP9 co-cultured MEPs and CMPs. M. Representative FACS plots of TER119 and CD41 expression of day 7 OP9 co-cultured wild type E16.5 MEPs. N. Representative FACS plots of CD11b, GR1, TER119 and CD41 expression of day 7 OP9 co-cultured wild type E16.5 CMPs. O. Proportion of TER119+ erythroid cells and CD41+ megakaryocyte cells in day 7 OP9 wild type E16.5 MEP co-cultures. P. Proportions of granulocytes/monocytes, erythroid cells and megakaryocytes in day 7 OP9 wild type E16.5 CMPs co-cultures. N = 5. (PDF) [file pgen.1007127.s005.pdf]

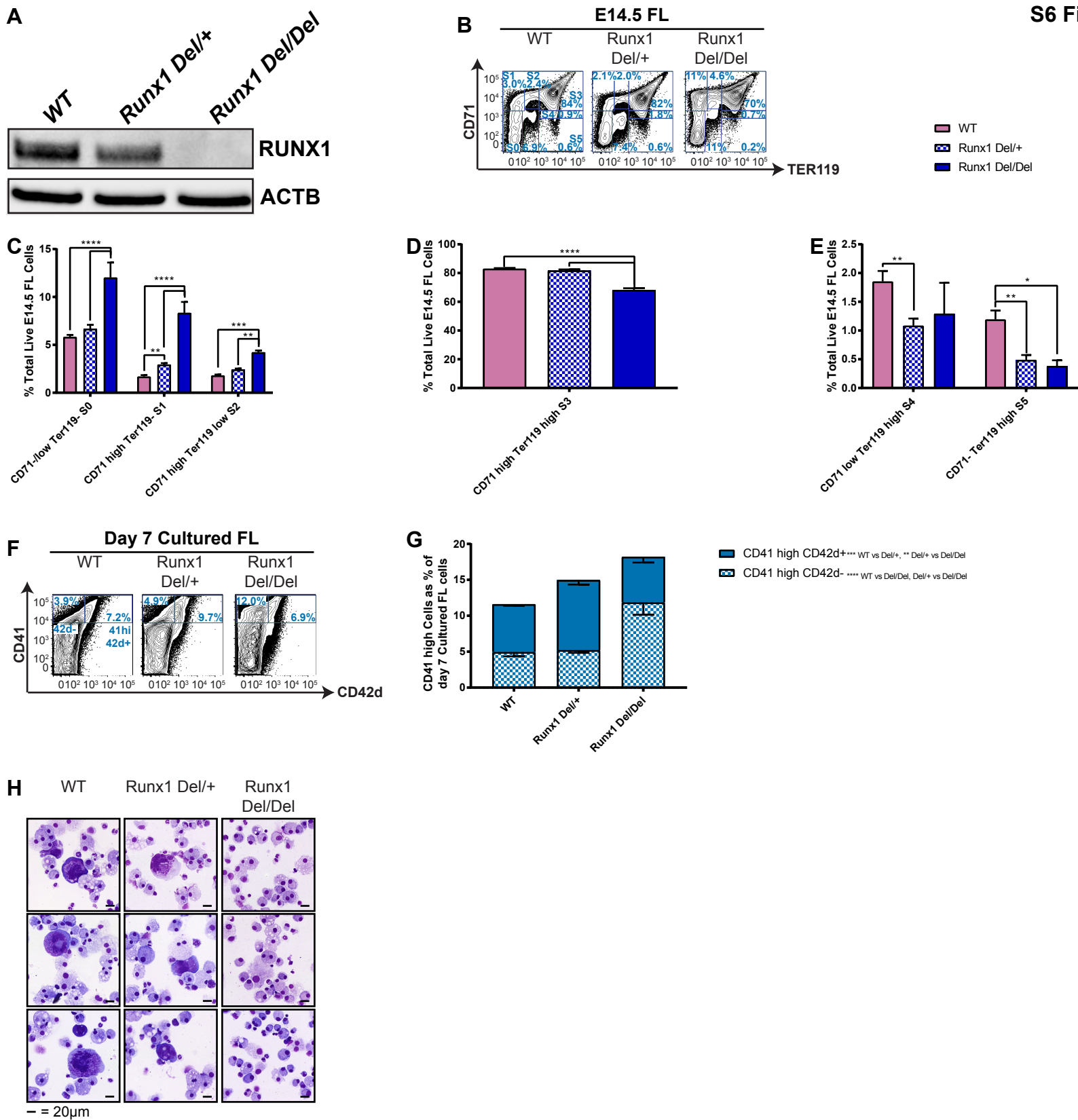

Supplement: S6 Fig — A. RUNX1/ACTINB Western blot of total protein extract from wild type, Runx1-del/+ and Runx1-del/del E14.5 fetal liver. Representative of 3 independent experiments. B-E. Characterization of erythroid lineage subsets S0-S5 in wild type, Runx1-del/+ and Runx1-del/del E14.5 fetal liver. B. Representative FACS plots of CD71/TER119 expression. C-E. Quantitation of S0 (CD71-/low TER119-), S1 (CD71high TER119-), S2 (CD71high TER119low) (C), S3 (CD71high TER119high) (D), S4 (CD71low TER119high) and S5 (CD71- TER119high) (E) erythroid populations. WT N = 10, Runx1-del/+ N = 9, Runx1-del/del N = 3. F-G. Characterization of CD41high CD42d- and CD41high CD42d+ megakaryocytes from day 7 total fetal liver cultures (wild type, Runx1-del/+ and Runx1-del/del). F. Representative FACS plots of CD41/CD42d expression. G. Quantitation of megakaryocytic CD41high CD42d- and mature megakaryocyte CD41high CD42d+ fractions. WT N = 10, Runx1-del/+ N = 9, Runx1-del/del N = 3. H. Morphologic analysis of day 7 cultured megakaryocytes, stained with May-Grünwald Giemsa reagent. (PDF) [file pgen.1007127.s006.pdf]

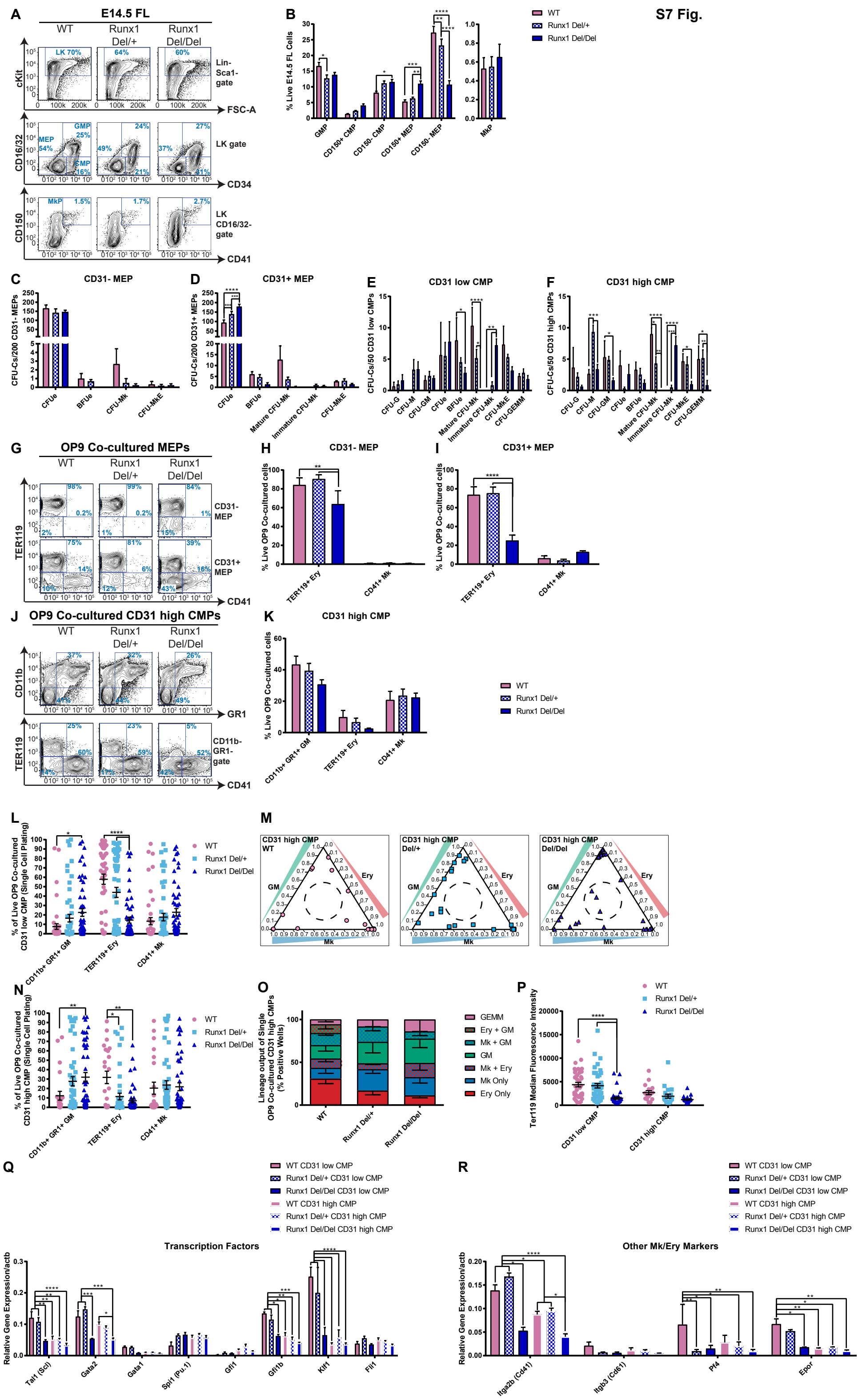

Supplement: S7 Fig — A-B. Characterization of myeloid progenitor populations in wild type, Runx1-del/+ and Runx1-del/del E14.5 FL. A. Representative FACS plots of MEP, CMP, GMP and MkP populations. B. Quantitation of myeloid hematopoietic progenitors. N = 6. C-F. Differential CFU-C activity of CD31- MEPs (C), CD31+ MEPs (D), CD31low CMPs (E) and CD31high CMPs (F). N = 5. G-I. Lineage output of bulk OP9 co-cultured wild type, Runx1-del/+ and Runx1-del/del E14.5 fetal liver CD31- and CD31+ MEPs. G. Representative FACS plots of TER119/CD41 expression. H-I. Quantitation of TER119+ erythroid and CD41+ megakaryocyte cells in CD31- MEP (H) and CD31+ MEP (I) co-cultures. N = 4. J-K. Lineage output of bulk OP9 co-cultured wild type, Runx1-del/+ and Runx1-del/del E14.5 fetal liver CD31high CMPs. J. Representative FACS plots of CD11b/GR1 and TER119/CD41 expression. K. Quantitation of CD11b+ GR1+ granulocyte/monocyte, TER119+ erythroid and CD41+ megakaryocyte cells in CD31high CMP co-cultures. N = 4. L-P. Lineage output of single OP9 co-cultured wild type, Runx1-del/+ and Runx1-del/del E14.5 fetal liver CMPs. L. Quantitation of CD11b+ GR1+ granulocyte/monocyte, TER119+ erythroid and CD41+ megakaryocyte populations in CD31low CMPs. M. Ternary plots displaying proportions of granulocyte/monocyte, erythroid and megakaryocyte cells in each positive well for CD31high CMPs. N. Quantitation of CD11b+ GR1+ granulocyte/monocyte, TER119+ erythroid and CD41+ megakaryocyte populations in CD31high CMP cultures. O. Proportions of unilineage (Ery, Mk and GM only) and multilineage (Mk + Ery, Mk + GM, Ery + GM and GEMM) wells derived from CD31high CMPs. P. TER119 Median Fluorescence Intensity of cells of TER119+ cells. N = 3. Q-R. Quantitative PCR analysis of the expression of selected RUNX1-target and/or hematopoietic lineage-associated genes in CD31low and CD31high CMPs. Q. Expression of RUNX1-associated transcription factors. R. Expression of other megakaryocytic and erythroid markers. N = 3 (PDF) [file pgen.1007127.s007.pdf]

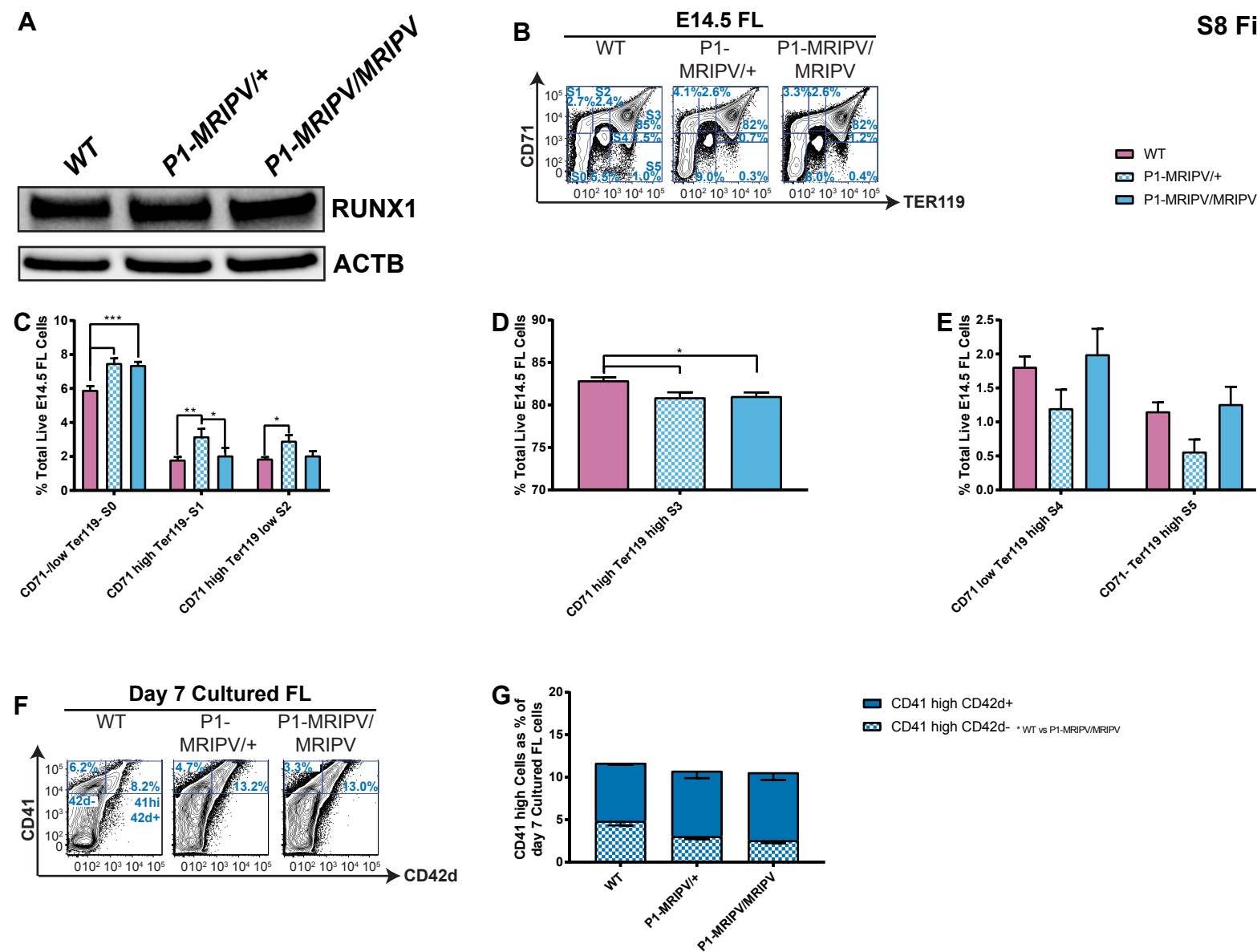

Supplement: S8 Fig — A. RUNX1/ACTINB Western blot of total protein extract from wild type, Runx1-P1-MRIPV/+ and Runx1-P1-MRIPV/MRIPV E14.5 fetal liver. Representative of 3 independent experiments. B-E. Characterization of erythroid lineage subsets S0-S5 in wild type, Runx1-P1-MRIPV/+ and Runx1-MRIPV/MRIPV E14.5 fetal liver. B. Representative FACS plots of CD71/TER119 expression. C-E. Quantitation of S0, S1 and S2 (C), S3 (D), and S4 and S5 (E) erythroid populations. WT N = 12, Runx1-P1-MRIPV/+ N = 9, Runx1-P1-MRIPV/MRIPV N = 9. F-G. Characterization of CD41high CD42d- and CD41high CD42d+ megakaryocytes from day 7 total fetal liver cultures (Runx1 wild type, P1-MRIPV/+ and P1-MRIPV/MRIPV). F. Representative FACS plots of CD41/CD42d expression. G. Quantitation of megakaryocyte CD41high CD42d- and mature megakaryocyte CD41high CD42d+ fractions. WT N = 12, Runx1-P1-MRIPV/+ N = 9, Runx1-P1-MRIPV/MRIPV N = 9. (PDF) [file pgen.1007127.s008.pdf]

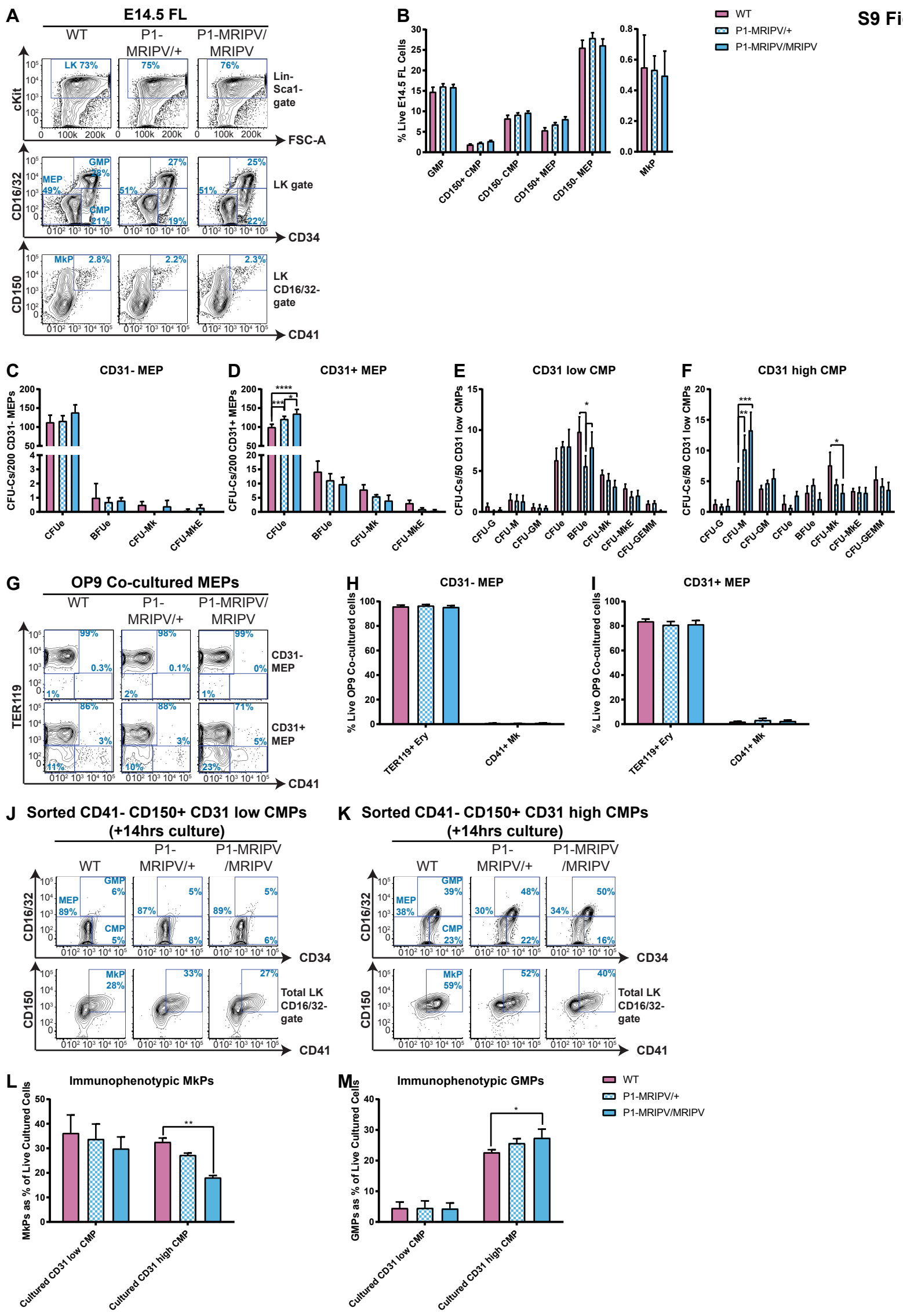

Supplement: S9 Fig — A-B. Characterization of myeloid progenitor populations in wild type, Runx1-P1-MRIPV/+ and Runx1-P1-MRIPV/MRIPV E14.5 fetal liver. A. Representative FACS plots of MEP, CMP, GMP and MkP populations. B. Quantitation of myeloid hematopoietic progenitors. N = 7. C-F. Differential CFU-C activity of CD31- MEPs (C), CD31+ MEPs (D), CD31low CMPs (E) and CD31high CMPs (F). N = 5. G-I. Lineage output of bulk OP9 co-cultured wild type, Runx1-P1-MRIPV/+ and Runx1-P1-MRIPV/MRIPV E14.5 fetal liver CD31- and CD31+ MEPs. G. Representative FACS plots of TER119/CD41 expression. H-I. Quantitation of TER119+ erythroid and CD41+ megakaryocyte cells in CD31- MEP (H) and CD31+ MEP (I) co-cultures. N = 5. J-M. Short-term (14 hours) differentiation of E14.5 wild type, Runx1-P1-MRIPV/+ and Runx1-P1-MRIPV/MRIPV CD31-/+ MEPs and CD31low/high CMPs in pro-myeloid liquid culture. J-K. Representative FACS plots of cultured CD31low CMPs (J) and CD31high CMPs (K). L-M. Proportions of immunophenotypic MkPs (L) and GMPs (M) in hematopoietic progenitor cultures. N = 3. (PDF) [file pgen.1007127.s009.pdf]
