# Supplementary material for: A novel prospective isolation of murine fetal liver progenitors to study in utero hematopoietic defects
Source: PLoS Genet. 2018 Jan 4;14(1):e1007127. doi: 10.1371/journal.pgen.1007127 (PMC5754050; doi:10.1371/journal.pgen.1007127)
Supplement: S1 Table — (DOCX) [file pgen.1007127.s010.docx]

**S1 Table. Primers used for mouse genotyping PCRs**

| **Mouse Line(s)** | **PCR** | **Oligonucleotide 1** | **Oligonucleotide 2** | **Oligonucleotide 3** | **PCR product sizes** |
| --- | --- | --- | --- | --- | --- |
| *P1-GFP::P2-hCD4*  *P1-GFP::P2-RFP* | *P1-GFP* | agttcaacccacagcataggcgg | atggtgatacaagggacatcttccc | aaaacccaaagagtgtcctccgcg | WT = 652bp  *P1-GFP* =413bp |
| *P1-MRIPV* | *P1-MRIPV* PCR 1 | agttcaacccacagcataggcgg | caaaaatgctgtctgaagccatcgtttcc | N/A | WT = 300bp  *P1-MRIPV* = No band |
|  | *P1-MRIPV* PCR 2 | agttcaacccacagcataggcgg | cctacggggatacgcatcgtttcc | N/A | WT = No band  *P1-MRIPV* = 305bp |
| *Runx1 flox::Vav1-Cre* | *Runx1 Flox/Del/WT* | gagtcccagctgtcaattcc | ggtgatggtcagagtgaagc | ccaagatagtccttaacggtcg | WT = 380bp  *Runx1 flox* = 450bp  *Runx1 Del* = 280bp |
|  | *Vav1-Cre* | caggttttggtgcacagtca | ggtgttgtagttgtccccact | N/A | WT = No band  *Vav1-Cre* = 390bp |
| *GFI1-GFP* | *GFI1-GFP* | cccttctctcagaactcagag | ggaaacgaggtggcttggag | gtcttgtagttgccgtcgtc | WT = 245bp  *GFI1-GFP* = 390bp |
| *GFI1b-GFP* | *GFI1b-GFP* | gcgcttgtcactcttagtcac | cctcaactccaaaggctagag | gctgttgtagttgtactccag | WT = 500bp  *GFI1b-GFP* = 390bp |
