# Supplementary material for: A novel prospective isolation of murine fetal liver progenitors to study in utero hematopoietic defects
Source: PLoS Genet. 2018 Jan 4;14(1):e1007127. doi: 10.1371/journal.pgen.1007127 (PMC5754050; doi:10.1371/journal.pgen.1007127)
Supplement: S2 Table — (DOCX) [file pgen.1007127.s011.docx]

**S2 Table. Details of Flow Cytometry Reagents**

| **Antibody­­** | **Conjugation** | **Clone** | **Reactivity** | **Host Species** | **Supplier** |
| --- | --- | --- | --- | --- | --- |
| CD3E | Biotin | 145-2C11 | Mouse | Armenian Hamster | ThermoFisher Scientific |
| CD4 | Biotin | GK1.5 | Mouse | Rat | ThermoFisher Scientific |
| CD4 | PE Cy7 | SK3 | Human | Mouse | ThermoFisher Scientific |
| CD4 | PE Alexa Fluor 610 | S3.5 | Human | Mouse | ThermoFisher Scientific |
| CD5 | Biotin | 53-7.3 | Mouse | Rat | ThermoFisher Scientific |
| CD8A | Biotin | 53-6.7 | Mouse | Rat | ThermoFisher Scientific |
| CD11B (MAC1) | APC eFluor 780 | M1/70 | Mouse | Rat | ThermoFisher Scientific |
| CD16/CD32 | PE Cy7, PerCPCy5.5, Alexa Fluor 700 | 93 | Mouse | Rat | ThermoFisher Scientific |
| CD24 | PE | 30-F1 | Mouse | Rat | ThermoFisher Scientific |
| CD29 | PE Cy7 | HMb1-1 | Mouse, rat | Armenian Hamster | ThermoFisher Scientific |
| CD31 | PE Cy7, Alexa Fluor 647 | 390 | Mouse | Rat | ThermoFisher Scientific |
| CD34 | FITC, eFluor 660 | RAM34 | Mouse | Rat | ThermoFisher Scientific |
| CD41 | eFluor 450, APC, PE Cy7 | MWReg30 | Mouse | Rat | ThermoFisher Scientific |
| CD42d | APC | 1C2 | Mouse | Rat | ThermoFisher Scientific |
| CD43 | APC | S7 | Mouse | Rat | BD Biosciences |
| CD44 | PE Cy7 | IM7 | Mouse, Human | Rat | ThermoFisher Scientific |
| CD45 | PerCPCy5.5, PE Cy7 | 30-F11 | Mouse | Rat | ThermoFisher Scientific |
| CD45R (B220) | Biotin | RA3-6B2 | Mouse, Human | Rat | ThermoFisher Scientific |
| CD48 | PE, APC | HM48-1 | Mouse | Armenian Hamster | ThermoFisher Scientific |
| CD55 | Alexa Fluor 647 | RIKO-3 | Mouse | Armenian Hamster | BioLegend |
| CD71 (Transferrin Receptor) | Biotin | R17217 | Mouse | Rat | ThermoFisher Scientific |
| CD105 (Endoglin) | PE | MJ7/18 | Mouse | Rat | ThermoFisher Scientific |
| CD117 (CKIT) | APC eFluor 780 | 2B8 | Mouse | Rat | ThermoFisher Scientific |
| CD117 (CKIT) | Brilliant Violet 421 | 2B8 | Mouse | Rat | BioLegend |
| CD127 | Biotin | A7R34 | Mouse | Rat | ThermoFisher Scientific |
| CD140a | PE | APA5 | Mouse | Rat | ThermoFisher Scientific |
| CD144 | Alexa Fluor 647 | BV13 | Mouse | Rat | ThermoFisher Scientific |
| CD150 | PE, PerCPCy5.5 | TC15-12F12.2 | Mouse | Rat | BioLegend |
| CD184 | APC | 2B11 | Mouse | Rat | ThermoFisher Scientific |
| CD202b (Tie2) | PE | TEK4 | Mouse | Rat | ThermoFisher Scientific |
| CD309 (FLK1) | PE Cy7 | Avas12a1 | Mouse | Rat | BioLegend |
| LY-6A/E (SCA1) | Biotin, PerCPCy5.5 | D7 | Mouse | Rat | ThermoFisher Scientific |
| LY-6G (GR1) | Biotin, PE Cy7 | RB6-8C5 | Mouse | Rat | ThermoFisher Scientific |
| TER119 | Biotin, PE | TER-119 | Mouse | Rat | ThermoFisher Scientific |
|  |  |  |  |  |  |
| **Reagent** | **Conjugation** |  |  |  | **Supplier** |
| Streptavidin | Pacific Orange, PE Cy7 |  |  |  | ThermoFisher Scientific |
| Hoechst 33258,  Pentahydrate  (bis-Benzimide) |  |  |  |  | ThermoFisher Scientific |

APC = Allophycocyanin; Cy = Cyanine; FITC = Fluorescein isothiocyanate; PE = Phycoerythrin; PerCP = Peridinin Chlorophyll Protein
