# Supplementary material for: A novel prospective isolation of murine fetal liver progenitors to study in utero hematopoietic defects
Source: PLoS Genet. 2018 Jan 4;14(1):e1007127. doi: 10.1371/journal.pgen.1007127 (PMC5754050; doi:10.1371/journal.pgen.1007127)
Supplement: S3 Table — (DOCX) [file pgen.1007127.s012.docx]

**S3 Table. Antibody combinations used for flow cytometric analysis and sorting (see S2 table for clone and supplier details)**

| **Panel (see Figure number)** | **Identified Populations** | **Primary Antibody/conjugate** | **Secondary Antibody**  **/conjugate** | **Other Reagents** |
| --- | --- | --- | --- | --- |
| LK CD150/CD55 Progenitors  (Fig 1A-B; S1B-F Fig) | FL CD55/CD150-expressing MEPs, CMPs, GMPs and MkPs | Lineage Cocktail: (anti-mouse CD3E/biotin, CD4/biotin, CD5/biotin, CD8A/biotin, B220/biotin, GR1/biotin, TER119/biotin, CD127/biotin)  Anti-mouse SCA1/biotin  Anti-mouse CKIT APC eFluor 780  Anti-mouse CD16/CD32/PE Cy7  Anti-mouse CD41/eFluor 450  Anti-mouse CD34/FITC  Anti-mouse CD150/PE  Anti-mouse CD55/Alexa Fluor 647 | Streptavidin  /Pacific Orange | Hoechst 33258  (Live/Dead  discrimination) |
| Myeloid OP9 Co-culture  (Fig 1F-I, 2D-G, 3D-G, 4F-I, 5D-H, 6D-G; S1G-H, S2C-F, S3C-F, S4I-J, S5E-H, S5M-P, S7G-P, S9G-I Fig) | Cultured CD41+ Mk, TER119+ Erythroid, CD11B+ GR1+ GM and CKIT+ Mast/HPCs | Anti-mouse CD41/APC  Anti-mouse TER119/PE  Anti-mouse CD11B/APC eFluor 780  Anti-mouse GR1/PE Cy7  Anti-mouse CKIT/Brilliant Violet 421 (Figure 1F-G, S1E-F only) |  | Hoechst 33258  (Live/Dead  discrimination) |
| *Runx1 P1-GFP::P2-hCD4* LK CD150 Progenitors  (Fig 2A-B, 3A-B) | FL *Runx1 P1-GFP::P2-hCD4*-expressing CD150+/- CMPs and MEPs | Lineage Cocktail  Anti-mouse SCA1/biotin  Anti-mouse cKit/APC eFluor 780  Anti-mouse CD16/CD32/PerCP Cy5.5  Anti-mouse CD41/eFluor 450  Anti-mouse CD34/eFluor 660  Anti-mouse CD150/PE  Anti-human CD4/PE Cy7 | Streptavidin  /Pacific Orange | Hoechst 33258  (Live/Dead  discrimination) |
| Cultured *Runx1 P1-GFP::P2-hCD4* MEPs and CMPs  (Fig 2H-I, 3H-I) | 14hr-cultured *Runx1 P1-GFP::P2-hCD4* MEPs and CMPs | Lineage Cocktail  Anti-mouse SCA1/biotin  Anti-mouse cKit/APC eFluor 780  Anti-mouse CD16/CD32/Alexa Fluor 700  Anti-mouse CD41/eFluor 450  Anti-mouse CD34/eFluor 660  Anti-mouse CD150/PerCPCy5.5  Anti-human CD4/PE Cy7 | Streptavidin  /Pacific Orange | Hoechst 33258  (Live/Dead  discrimination) |
| *Runx1 P1-GFP::P2-hCD4* LK CD150 CD45/CD31/CD48 Progenitors  (Fig 4A, S5A-B, S5I-J Fig) | CD45/CD31/CD48 expression in FL *Runx1 P1-GFP::P2-hCD4*-LK progenitors | Lineage Cocktail  Anti-mouse SCA1/biotin  Anti-mouse cKit/APC eFluor 780  Anti-mouse CD16/CD32/Alexa Fluor 700  Anti-mouse CD41/eFluor 450  Anti-mouse CD34/eFluor 660  Anti-human CD4/PE Alexa Fluor 610  Anti-mouse CD31/PE Cy7  Anti-mouse CD150/PE or PerCPCy5.5  Anti-mouse CD45 PerCPCy5.5 or anti-mouse CD48 PE | Streptavidin  /Pacific Orange | Hoechst 33258  (Live/Dead  discrimination) |

| LK CD150+ CD31 Progenitors  (Fig 4C, 5A-C, 6A-C; S7A-B, S9A-B Fig) | FL CD150/CD31-expressing MEPs and CMPs | Lineage Cocktail  Anti-mouse SCA1/biotin  Anti-mouse cKit/APC eFluor 780  Anti-mouse CD16/CD32/PE Cy7  Anti-mouse CD41/eFluor 450  Anti-mouse CD34/FITC  Anti-mouse CD31/Alexa Fluor 647  Anti-mouse CD150/PE  Anti-mouse CD45 PerCPCy5.5 | Streptavidin  /Pacific Orange | Hoechst 33258  (Live/Dead  discrimination) |
| --- | --- | --- | --- | --- |
| LSK HSPCs (S1I Fig) | FL LSK CD48/CD150-expressing HSPCs | Lineage Cocktail  Anti-mouse cKit/APC eFluor 780  Anti-mouse SCA1/PerCPCy5.5  Anti-mouse CD150/PE  Anti-mouse CD48/APC | Streptavidin  /Pacific Orange | Hoechst 33258  (Live/Dead  discrimination) |
| Cultured FL LSK HSPCs and yolk sac cells (S1K-N) | Uncultured and pro-myeloid cultured (20 hours+) FL LSK HSPCs and yolk sac cells | Lineage Cocktail  Anti-mouse cKit/APC eFluor 780  Anti-mouse SCA1/PerCPCy5.5  Anti-mouse CD150/PE  Anti-mouse CD48/APC  Anti-mouse CD34/FITC  Anti-mouse CD41/eFluor 450  Anti-mouse CD16/CD32/Alexa Fluor 700 | Streptavidin  /Pacific Orange | Hoechst 33258  (Live/Dead  discrimination) |
| *Runx1 P1-GFP::P2-hCD4* Correlating Markers Screen (S4C Fig) | Total FL *Runx1 P1-GFP::P2-hCD4*-expressing cells plus panel of potential correlating cell surface markers | Anti-human CD4/PE Alexa Fluor 610  Anti-mouse CD105/PE or anti-mouse Tie2/PE or anti-mouse CD48/PE or anti-mouse CD24/PE or anti-mouse CD140a/PE  Anti-mouse CD45/PE Cy7 or anti-mouse CD309/PE Cy7 or anti-mouse CD44/PE Cy7 or anti-mouse CD29/PE Cy7  Anti-mouse CD11b/APC or anti-mouse CD144/Alexa Fluor 647 or anti-mouse CD31/Alexa Fluor 647 or anti-mouse CD43/APC or anti-mouse CD184/APC |  | Hoechst 33258  (Live/Dead  discrimination) |
| LK CD150+ CD48 Progenitors  (S4E Fig) | FL CD150/CD48-expressing MEPs and CMPs | Lineage Cocktail  Anti-mouse SCA1/biotin  Anti-mouse cKit/APC eFluor 780  Anti-mouse CD16/CD32/PE Cy7  Anti-mouse CD41/eFluor 450  Anti-mouse CD34/FITC  Anti-mouse CD48/APC  Anti-mouse CD150/PE  Anti-mouse CD45/PerCPCy5.5 | Streptavidin  /Pacific Orange | Hoechst 33258  (Live/Dead  discrimination) |

| *Gfi1-GFP* and *Gfi1b-GFP* LK CD150 Progenitors  (S4F-G Fig) | FL *Gfi1-GFP* or *Gfi1b-GFP*-expressing CD150/CD31 MEPs and CMPs | Lineage Cocktail  Anti-mouse SCA1/biotin  Anti-mouse cKit/APC eFluor 780  Anti-mouse CD16/CD32/PerCPCy5.5  Anti-mouse CD41/eFluor 450  Anti-mouse CD34/eFluor 660  Anti-mouse CD31/PE Cy7  Anti-mouse CD150/PE | Streptavidin  /Pacific Orange | Hoechst 33258  (Live/Dead  discrimination) |
| --- | --- | --- | --- | --- |
| Cultured CD150+ CD31 MEPs and CMPs (S4K-N, S9J-M Fig) | 14hr-cultured MEPs and CMPs expressing CD150/CD31 | Lineage Cocktail  Anti-mouse SCA1/biotin  Anti-mouse cKit/APC eFluor 780  Anti-mouse CD16/CD32/Alexa Fluor 700  Anti-mouse CD41/eFluor 450  Anti-mouse CD34/FITC  Anti-mouse CD31/Alexa Fluor 647  Anti-mouse CD150/PerCPCy5.5  Anti-mouse CD45/PE Cy7 | Streptavidin  /Pacific Orange | Hoechst 33258  (Live/Dead  discrimination) |
| FL Erythroid Lineage Populations  (S6B-E, S8B-E Fig) | S0 (CD71^-/low^TER119^-^)  S1 (CD71^high^TER119^-^)  S2 (CD71^high^TER119^low^)  S3 (CD71^high^TER119^high^)  S4 (CD71^low^TER119^high^)  S5 (CD71^-^TER119^high^) | Anti-mouse CD71/biotin  Anti-mouse TER119/PE | Streptavidin/PE Cy7 | Hoechst 33258  (Live/Dead  discrimination) |
| Cultured megakaryocytes  (S6F-G, S8F-G Fig) | Day 7 cultured FL megakaryocytes expressing CD41/CD42d | Anti-mouse CD41/PE Cy7  Anti-mouse CD42d/APC |  | Hoechst 33258  (Live/Dead  discrimination) |
