# Supplementary material for: A novel prospective isolation of murine fetal liver progenitors to study in utero hematopoietic defects
Source: PLoS Genet. 2018 Jan 4;14(1):e1007127. doi: 10.1371/journal.pgen.1007127 (PMC5754050; doi:10.1371/journal.pgen.1007127)
Supplement: S4 Table — (DOCX) [file pgen.1007127.s013.docx]

**S4 Table. Primers and probes used for qPCR**

| **Target Gene** | **Primer 1** | **Primer 2** | **Roche Universal ProbeLibrary Probe ID** |
| --- | --- | --- | --- |
| Actb | tgacaggatgcagaagaaga | cgctcaggaggagcaatg | 106 |
| Tal1 (Scl) | gctcgcctcactaggcagt | cttcacccggttgttgttg | 60 |
| Gata2 | cacaagatgaatggacagaacc | acaggtgcccgctcttct | 75 |
| Gata1 | ccctgaactcgtcataccact | gaacactggggttgaacctg | 83 |
| Spi1 (Pu.1) | ggagaagctgatggcttgg | caggcgaatctttttcttgc | 94 |
| Gfi1 | atgtgcggcaagaccttc | acagtcaaagctgcgttcct | 1 |
| Gfi1b | tacccctgccagttctgtg | cttgtggggcttctcacct | 31 |
| Klf1 | caagagctcgcacctcaag | gagcgaacctccagtcaca | 68 |
| Fli1 | agaccatgggcaagaacact | gccccaggatctgataagg | 20 |
| Itga2b (Cd41) | tgctgctgaccctgctagt | gtcgattccgcttgaagaag | 97 |
| Itgb3 (Cd61) | gtgggagggcagtcctcta | caggatatcaggacccttgg | 31 |
| Pf4 | catctcctctgggatccatct | ccattcttcagggtggctat | 9 |
| Epor | gtcctcatctcgctgttgct | atgccaggccagatcttct | 56 |
